# Salt Stress Affects Plastid Ultrastructure and Photosynthetic Activity but Not the Essential Oil Composition in Spearmint (*Mentha spicata* L. var. *crispa* “Moroccan”)

**DOI:** 10.3389/fpls.2021.739467

**Published:** 2021-10-29

**Authors:** Roumaissa Ounoki, Ferenc Ágh, Richard Hembrom, Renáta Ünnep, Bernadett Szögi-Tatár, Andrea Böszörményi, Katalin Solymosi

**Affiliations:** ^1^Department of Plant Anatomy, ELTE Eötvös Loránd University, Budapest, Hungary; ^2^Department of Pharmacognosy, Semmelweis University, Budapest, Hungary; ^3^Neutron Spectroscopy Department, Center for Energy Research, Budapest, Hungary

**Keywords:** chlorophyll, chloroplast, granum, polyethylene glycol (PEG), small-angle neutron scattering (SANS), swelling, transmission electron microscope (TEM), vegetative propagation

## Abstract

High levels of soil salinity affect plant growth, reproduction, water and ion uptake, and plant metabolism in a complex manner. In this work, the effect of salt stress on vegetative growth, photosynthetic activity, and chloroplast ultrastructure of spearmint (*Mentha spicata* L. var. *crispa* “Moroccan”) was investigated. After 2 weeks of low concentration treatments (5, 25, and 50 mM NaCl) of freshly cut shoots, we observed that the stem-derived adventitious root formation, which is a major mean for vegetative reproduction among mints, was completely inhibited at 50 mM NaCl concentration. One-week-long, high concentration (150 mM NaCl) salt stress, and isosmotic polyethylene glycol (PEG) 6000 treatments were compared in intact (rooted) plants and freshly cut, i.e., rootless shoots. Our data showed that roots have an important role in mitigating the deleterious effects of both the osmotic (PEG treatment) and specific ionic components of high salinity stress. At 50 mM NaCl or above, the ionic component of salt stress caused strong and irreversible physiological alterations. The effects include a decrease in relative water content, the maximal and actual quantum efficiency of photosystem II, relative chlorophyll content, as well as disorganization of the native chlorophyll-protein complexes as revealed by 77 K fluorescence spectroscopy. In addition, important ultrastructural damage was observed by transmission electron microscopy such as the swelling of the thylakoid lumen at 50 mM NaCl treatment. Interestingly, in almost fully dry leaf regions and leaves, granum structure was relatively well retained, however, their disorganization occurred in leaf chloroplasts of rooted spearmint treated with 150 mM NaCl. This loss of granum regularity was also confirmed in the leaves of these plants using small-angle neutron scattering measurements of intact leaves of 150 mM NaCl-stressed rooted plants. At the same time, solid-phase microextraction of spearmint leaves followed by gas chromatography and mass spectrometry (GC/MS) analyses revealed that the essential oil composition of spearmint was unaffected by the treatments applied in this work. Taken together, the used spearmint cultivar tolerates low salinity levels. However, at 50 mM NaCl concentration and above, the ionic components of the stress strongly inhibit adventitious root formation and thus their clonal propagation, and severely damage the photosynthetic apparatus.

## Introduction

Spearmint is a perennial herbaceous medicinal plant from the Lamiaceae family. The plants that belong to this family and genus are a rich source of secondary metabolites like terpenoids and polyphenols which have strong biological effects (Gulluce et al., 2007; Bimakr et al., 2011; Mahendran and Rahman, 2020). Therefore, such plants are often used as medicinal and aromatic plants or for culinary purposes.

Mint has been used by humans to disinfect homes after infectious disease or death for ages, and this way it came to represent cleanliness. Studies confirmed that the leaves of spearmint (*Mentha spicata L. var. crispa)* have anti-fungal, anti-microbial, anti-inflammatory, anti-tumor, and antioxidant activities (Mata et al., 2007; Sokovi et al., 2009; Lixandru et al., 2010; Guimarães et al., 2011; Chrysargyris et al., 2017; Salehi et al., 2018). Furthermore, its essential oil used to be an official drug in the Hungarian Pharmacopeia under the name *Aetheroleum menthae crispae*. In folk medicine, spearmint has been attributed a variety of therapeutic properties, including the prevention of chemotherapy-induced nausea and vomiting (CINV) and the treatment of respiratory and digestive troubles (Karousou et al., 2007; Cakilcioglu et al., 2011).

Spearmint essential oil is widely used in several products as an aromatic or fragrance agent such as medications and sweets, as well as chewing gum, toothpaste, and mouthwashes, and is an important compound of ecological pesticides and antimicrobial agents (Chrysargyris et al., 2017). Spearmint leaves and rarely essential oils are used to flavor teas, stews, and soups with the dried leaves used to preserve season meat such as lamb, fish, chicken, and vegetables such as rice (Mata et al., 2007; Igoumenidis et al., 2016; Salehi et al., 2018). Spearmint is also used as an ingredient in a variety of mixed drinks, including the mojito, and the mint julep.

The major constituent of spearmint essential oil is carvone, which provides spearmint its characteristic scent (Kokkini et al., 1995). In addition, spearmint essential oil also contains noteworthy concentrations of limonene and 1,8-cineole (Cirlini et al., 2016; Chrysargyris et al., 2017). Carvone is reported to inhibit bacterial growth and has fungicide and insect repellent properties (Oosterhaven et al., 1995).

Mint essential oil is among the 10 most commercialized essential oils. Moreover, spearmint is one of the most valuable flavors listed after vanilla and citrus (Chrysargyris et al., 2017). Therefore, spearmint is widely cultivated in all parts of the world and there is a growing demand for spearmint cultivation. The decrease in quality of arable land due to salinization, accumulation of heavy metals and organic pollutants, projected increase in temperature and erratic precipitation patterns represent a huge challenge to mankind. High soil salinity and high salt concentration in water available for irrigation effect and limit agricultural production in several areas worldwide including countries in the Mediterranean Region and the Sahara, e.g., Morocco and Algeria (Sarmoum et al., 2019), where mint production for pharmaceutical and food purposes is traditionally and economically important (El Hassani, 2020). Previous literature data indicated that wild mint is sensitive or moderately sensitive to salinity (Warrance et al., 2002). Peppermint is also highly sensitive to soil salinity, as it cannot maintain normal growth, biomass, and essential oil production in soils containing more than 75 mM NaCl (0.3%). Therefore, the breeding of salinity tolerant peppermint cultivars is important as well as understanding salinity stress induced cellular responses (Li et al., 2015). The effect of salt stress on photosynthesis and essential oil composition were studied in peppermint and other mint species (Aziz et al., 2008; Oueslati et al., 2010; Kasrati et al., 2014; Li et al., 2015; Yu et al., 2015). However, only one paper studied spearmint in the said respect (Chrysargyris et al., 2019) but it did not use a local, Moroccan cultivar. Data showed decreased growth, chlorophyll content, and altered essential oil profile under salt stress, but no 77 K fluorescence emission analyses and ultrastructural observations were carried out in these studies. Therefore, in this work, we wanted to investigate whether spearmint could be cultivated under salt stress conditions and how its photosynthetic tissues and especially the organization and structure of its photosynthetic apparatus were affected by salinity.

Soil salinity is expected to affect the world more severely and extensively in the coming years. Increasing salinity in water resources is also associated with increasing salinity in the soil (McFarlane and Williamson, 2002). High soil salinity influences plant growth and productivity in a complex manner. It can trigger both hyperionic and hyperosmotic stress in plants (Hasegawa et al., 2000; Pagter et al., 2009; Isayenkov and Maathuis, 2019; Wani et al., 2020). Under salt stress conditions, photosynthetic parameters have been shown to be suppressed in a variety of plant species (James et al., 2002, 2006; Chaves et al., 2009; Bose et al., 2017). However, there are contradictions in the literature about the effect of salt stress on plastid structure. In many studies, the swelling of the thylakoid lumen and disorganization of the thylakoid system in chloroplasts was observed under salt stress (Salama et al., 1994; Yamane et al., 2008, 2012; Omoto et al., 2010; Evelin et al., 2013; Gao et al., 2015a; Goussi et al., 2018). Salt stress has an effect on chloroplast and thylakoid ion homeostasis (Bose et al., 2017), as well as on the lipid bilayer membrane, its composition and stability. Furthermore, by increasing lipid peroxidation, salt stress can cause membrane damage (Miller et al., 2010; Yamane et al., 2012; Suo et al., 2017). Increased number and size of plastoglobules have often been related to various stresses and were also observed under salt stress in susceptible species (Hernández et al., 1995; Solymosi and Bertrand, 2012; Acosta-Motos et al., 2017). It is assumed that plastoglobules are related to salt tolerance or may be related to degradation or aging processes during stress. Starch accumulation may be also affected by salinity stress (de Morais et al., 2019).

The above ultrastructural observations, especially those reporting thylakoid swelling, were reported in leaf samples prepared using conventional chemical fixation (using aqueous, ionic buffers), dehydration, embedding, and subsequent ultrathin sectioning, contrasting, and analysis by transmission electron microscopy (TEM). Small-angle neutron scattering (SANS) is another non-invasive method that also provides information about the ultrastructure of thylakoid membranes, i.e., their repeat distance, RD, and periodic order, averaged over the entire sample volume in the neutron beam *in vitro* (Nagy et al., 2013). During the sample preparation for electron microscopy, fixation or other artifacts related to osmotic shock or imbalance may occur.

Therefore, we wanted to develop model plant systems using living, intact, or almost intact plants in which the effects of salt stress on chloroplast structure could be compared critically using SANS and TEM. In this work, our objective was to check whether the effects of salt stress can be studied and compared in intact plants with these two methods yielding novel ultrastructural data. In addition, we aimed to investigate salinity stress in spearmint and compare the effects of ionic and osmotic components of salt stress on the essential oil composition, photosynthetic activity, and chloroplast structure in seedlings treated with various NaCl concentrations through the root or in a model system through the shoot.

## Materials and Methods

### Plant Material and Treatment Conditions

**Figure 1:**
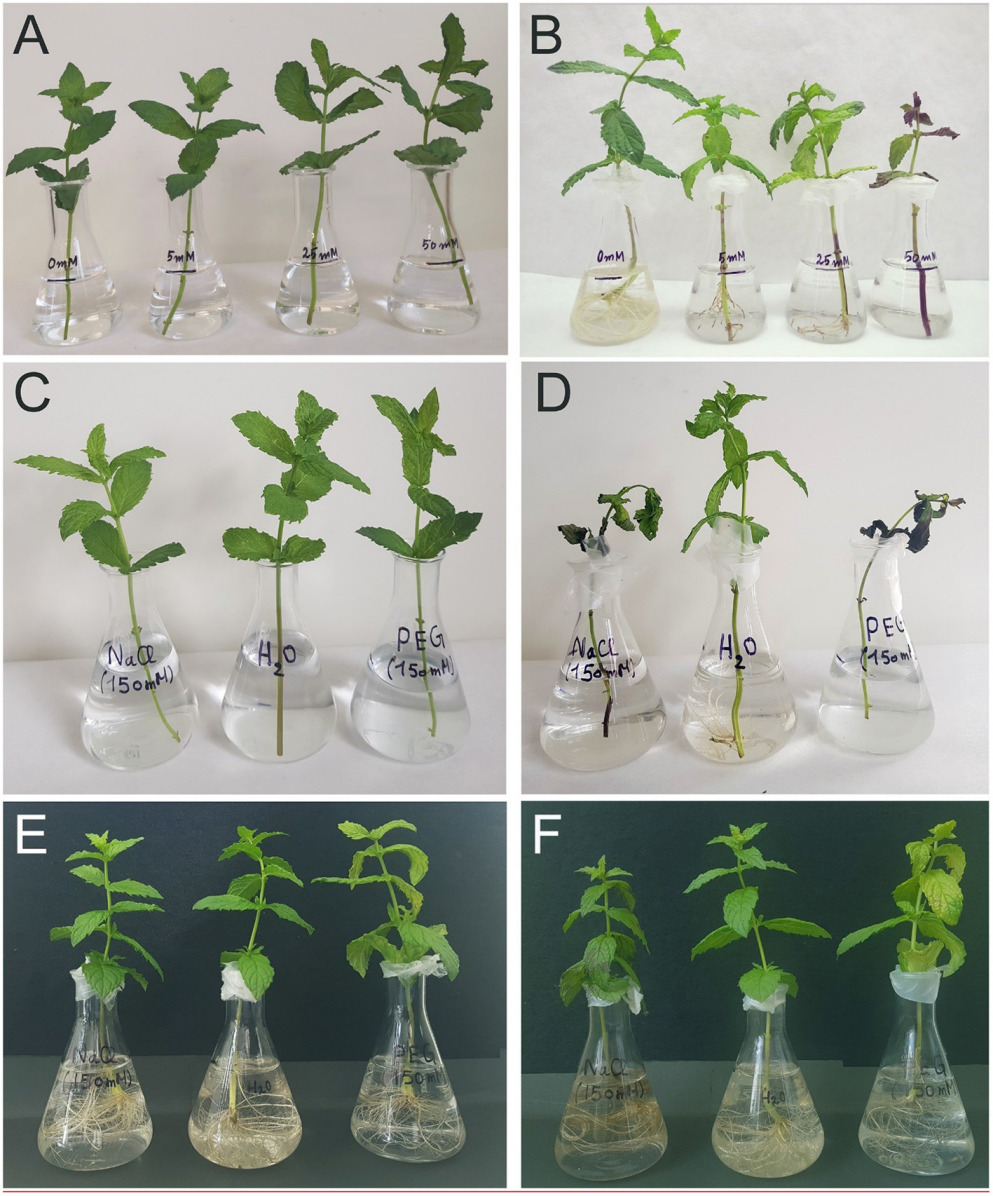
The phenotype of typical spearmint plants used for the various experiments in this work before **(A,C,E)** and after **(B,D,F)** of the applied treatments. **(A,B)** Adventitious root formation on freshly cut spearmint shoots exposed to 0, 5, 25, or 50 mM of NaCl (in distilled water) for 2 weeks. Please note that in the solution containing 50 mM NaCl concentration, no roots were formed **(B)**. **(C–F)** Freshly cut, i.e., rootless **(C,D)** or rooted **(E,F)** spearmint shoots treated with 0 mM NaCl (control, distilled water), 150 mM NaCl (in distilled water), and polyethylene glycol (PEG-6000, in distilled water, with equal osmolarity to the 150 mM NaCl solution) for 1 week. After positioning the plants **(A,C)**, the Erlenmeyer flasks were carefully covered with parafilm to avoid evaporation **(B,D–F)**.

Potted spearmint (*Mentha spicata* L. var. *crispa* “Moroccan”) plants were purchased from Etnoflóra company (Budapest, Hungary). Offsprings of the same clone were used throughout the experiments. Spearmint has a decussate leaf arrangement. Our measurements were performed using generally the third and fourth decussate leaf pairs on the aerial parts of the plants (numbering started from the shoot tip, and refers to the leaf pairs located at the third and fourth nodes), except for SANS measurements, where the upper part of the whole plant (first four leaf pairs and shoot tip) was measured. For essential oil determination, third, fourth, and fifth leaf pairs were used. We investigated the effect of salt stress on spearmint using two experimental setups. In the first experiment, we applied low concentrations of NaCl (VWR International Ltd., Hungary) stress to freshly cut 15- to 20-cm-long mint shoots for 14 days. During treatment, the shoots were immersed into distilled water supplemented with NaCl to reach 0, 5, 25, and 50 mM NaCl final concentrations (Figures 1A,B). In the second experiment, stronger salt stress (150 mM NaCl dissolved in distilled water) was applied for 7 days to the 15–20-cm-long freshly cut shoots (Figures 1C,D) and to rooted plants kept in glass Erlenmeyer flasks (Figures 1E,F). Flasks were closed by parafilm to minimize the evaporation of the solutions (Figure 1). In this high concentration setting, we wanted to compare the osmotic and ionic components of salt stress, therefore, a non-ionic component, polyethylene glycol (PEG-6000, Duchefa Biochemie, Netherlands) was used. Its amount was determined using Gonotec Osmomat 3000 basic osmometer (Roebling, Germany) and adjusted so that the osmolarity of the resulting solution was the same as that of the 150 mM NaCl solution (283 mOsm). The plants were kept at room temperature (25–26°C) and natural light conditions (~100–150 μmol s^−1^ m^−2^ photon flux density, 12:12 h photoperiod). The relative humidity of the air varied from 25 to 30%.

### Fluorescence Spectroscopy at 77K

One leaf segment per treated plant was placed in a glass tube and then immersed in liquid nitrogen. Fluorescence emission spectra at 77K were recorded using a FluoroMax 3 spectrofluorometer (Jobin Yvon-Horiba, France) with samples being immersed in a liquid nitrogen-containing dewar. The excitation wavelength was 440 nm, the integration time was 0.2 s, and the excitation and emission slits were set to 2 and 5 nm, respectively. Three spectra were recorded and automatically averaged for each sample. Emission signals were corrected for the wavelength-dependent sensitivity of the detection; when necessary, baseline correction, which eliminates light scattering effects, and 3-point and 5-point linear smoothing were also performed using the SPSERV V3.14 program (copyright: C. Bagyinka, Institute of Biophysics, Biological Research Center of the Hungarian Academy of Science, Hungary). Samples were collected from three independent repetitions and from different plants (*n* = 3–11). Due to differences in the size of the measured leaf segments and their geometry during measurement, the spectra were normalized to their maxima and these normalized spectra were averaged and are shown.

### Leaf Chlorophyll Content

A SPAD-502 Plus Chlorophyll Meter® (Konica Minolta, Japan) was used to estimate leaf chlorophyll content. Samples were collected from three independent experiments with two to four repetitions (*n* = 6–12) from different plants (third and fourth leaf pairs were measured on each plant, two to three different SPAD readings were taken per leaf), then average SPAD chlorophyll readings were calculated in each case as indicated in the Figure and Table legends (*n* = 80–147).

### Relative Water Content (RWC)

Plant water status was assessed by determining leaf relative water content (RWC) (González and González-Vilar, 2001). Measurements were carried out in three or four independent experiments, with three to eight biological replicates in each experiment. The replicates contained three to four leaf discs of 3 mm diameter from one leaf. RWC was estimated as [(FW - DW)/(TW – DW)] × 100, where FW is the fresh weight of the discs, TW is the turgid weight after overnight rehydration of the discs, by floating in distilled water at 4°C. The dry weight (DW) of the discs was obtained after oven-drying at 80°C until a constant weight was achieved.

### Photosynthetic Activity

The photosynthetic activity of the various samples was determined using a FluorPen FP 100 (Photon Systems Instruments, Czech Republic) portable instrument by recording fast chlorophyll fluorescence induction kinetics in light-adapted samples (“Qy light,” equivalent to F_v_‘/F_m_’) and also after 20 min of dark adaptation (“Qy dark,” equivalent to F_v_/F_m_), which parameters thus represent the actual and the maximal quantum efficiency of photosystem II, respectively (Björkman and Demmig, 1987). Measurements were carried out in at least three independent experiments, with 6–12 biological replicates in each experiment. From different plants, third and fourth leaf pairs were measured and 2 Qy measurements were taken per leaf. Average Qy data were calculated in each case as indicated in the Figure and Table legends (*n* = 16–36).

### Transmission Electron Microscopy (TEM)

Leaf sections (1 × 1 mm) were cut in 2.5% (v/v) glutaraldehyde, from the central parts of leaf blades of the treated leaf segments, with major veins being avoided. After sampling, leaf pieces were fixed in glutaraldehyde for at least 3 h, then post-fixed in 1% OsO_4_ (w/v) for 2 h. Fixatives were buffered with 70 mM Na_2_HPO_4_-KH_2_PO_4_ pH 7.2, and this solution was used three times for 15 min in order to wash out the excess of the fixatives from the tissues after each fixation step. After dehydration in an alcohol series, samples were embedded in Durcupan ACM resin (Fluka, Buchs, Switzerland). Ultrathin sections (70 nm) were prepared on a Reichert Jung ultramicrotome (Reichert Jung AG, Austria) and were stained with uranyl acetate and Reynold's lead citrate. Transmission electron microscopic (TEM) analyses were carried out with a JEOL JEM 1011 (JEOL Ltd., Japan) TEM at 80 kV accelerating voltage. Digital images were taken using the Olympus Morada CCD camera (Olympus Optical Co. Ltd., Japan). Fast Fourier transformation (FFT) on the selected region of interest of particular micrographs was performed using ImageJ (NIH, US) software to determine the granum RD values on them according to Ünnep et al. (2014). The number of randomly chosen chloroplasts was 30–35, and the RD values were averaged from 147 to 170 randomly chosen grana measured in them.

### Small-Angle Neutron Scattering (SANS)

Small-angle neutron scattering measurements were carried out at the “Yellow Submarine” SANS instrument of the Budapest Neutron Center (BNC, Budapest, Hungary). The sample-to-detector distance, the collimation distance, and the wavelength were set to 5.6, 4.5 m, and 5.76 Å, respectively. Intact rooted plants were placed on the sample holder. The acquisition time was 4 h, wherein consecutive profiles were then averaged to improve the relatively low S/N ratio. Three independent biological replicates were measured, and raw data were treated with the BerSANS program (Keiderling, 2002). The RD values (RD = 2π/q^*^, q^*^ is the center position of the Bragg peak) of granal thylakoid membrane comprised the widths of the lumen, of the interthylakoidal aqueous space, and twice the width of the membrane (Nagy and Garab, 2020). The values were obtained by fitting a linear combination of a constant, a power and a Gauss function on the radially averaged values. For further details, see (Füzi et al., 2017; Ünnep et al., 2020).

### Solid-Phase Microextraction (SPME) of the Essential Oil

Pooled intact and fresh third and fourth leaf pairs (0.5–1 g fresh mass) were used for the essential oil analyses. Samples were put into vials (20 ml headspace) sealed with a silicon/polytetrafluoroethylene septum prior to the static headspace solid-phase microextraction (sHS-SPME). A CTC Combi PAL (CTC Analytics AG, Switzerland) automatic multipurpose sampler was used for the sample preparation by applying the static headspace solid-phase microextraction (sHS-SPME) technique using a 65 mM StableFlex carboxene/polydimethylsiloxane/divinylbenzene (CAR/PDMS/DVB) SPME fiber (Supelco, US). After 5 min incubation at 100°C, the fiber was exposed to the headspace of the 20-ml vial containing the sample for 10 min at 100°C in order to obtain extraction. After this, the fiber was immediately transferred to the injector port of the gas chromatograph/mass spectrometer (GC/MS) and desorbed for 1 min at 250°C. The splitless mode was used for injections. In all cases, the SPME fiber was cleaned and conditioned in a Fiber Bakeout Station in a pure nitrogen atmosphere at 250°C for 15 min. SPME gas chromatography/mass spectrometry (SPME-GC/MS) analysis was performed after this.

### Gas Chromatography-Mass Spectrometry (GC/MS)

An Agilent 6890N/5973N GC/MSD (Agilent, US) system equipped with a Supelco (Sigma-Aldrich, US) SLB-5MS capillary column (30 m × 250 μm × 0.25 μm) was used for the measurements. According to the program that was used, the temperature of the GC oven increased from 60°C (3 min isothermal) to 250°C at 8°C/min (1 min isothermal). The carrier gas (high purity helium at 6) was applied at 1 ml/min (37 cm/s) in constant flow mode. Detection was obtained using a mass selective detector (MSD) equipped with a quadrupole mass analyzer. The detector was operated in electron ionization mode at 70 eV in full scan mode (41–500 amu at 3.2 scan/s). MSD ChemStation D 0.02.00.275 software (Agilent, US) was used for data analysis. Compounds were identified by comparing retention data and recorded spectra with literature data, and the NIST 2 library (NIST, US). Zone averaged data were used for percentage analyses. Standard errors are calculated from three or five independent biological replicates as indicated, measured in the same period of the year on the same clone.

### Statistical Analyses

Statistical analyses (normality test, ANOVA, and *post hoc* tests) were performed using GraphPad Prism 8 (GraphPad Software, US). In the case of data following normal distribution, and significant differences detected by one-way ANOVA, Tukey-Kramer multiple comparisons test was used as a *post hoc* test. Some data did not follow a normal distribution, in this case, the Kruskal-Wallis non-parametric ANOVA test was performed, followed by Dunn's multiple comparisons test as a *post hoc* test. Significant differences were labeled with different letters. For all data, *P* < 0.05 was considered significant.

## Results

Salinity affects an increasing amount of arable land, this way areas suitable for agricultural production are continuously diminishing. Therefore, we primarily wanted to investigate whether spearmint could be cultivated and propagated under low concentration salt stress conditions.

### The Effect of Low Concentration Salt Stress Treatments on Spearmint

In the case of mint species, adventitious roots produced on the rhizome or the lower nodes of the stem are often playing a major role in the vegetative reproduction and clonal growth of the plants. These roots were produced both during normal development and after injuries. In the latter case, differentiated tissues were regaining their meristematic function *via* dedifferentiation and subsequently produced a new root tip. In this work, we first checked in a model system whether adventitious root formation or chloroplast structure, photosynthesis, and essential oil composition were influenced by low levels of salinity. Therefore, we exposed freshly cut spearmint shoots to low levels (0, 5, 25, or 50 mM) of NaCl for 2 weeks (Figure 1).

We observed normal adventitious root formation in 0, 5, and 25 mM NaCl solutions, while no roots were observed in the case of the shoots immersed into 50 mM NaCl (compare Figures 1A,B). These data showed that relatively low NaCl concentrations readily inhibit adventitious root formation, an important factor in spearmint vegetative reproduction.

**Figure 2:**
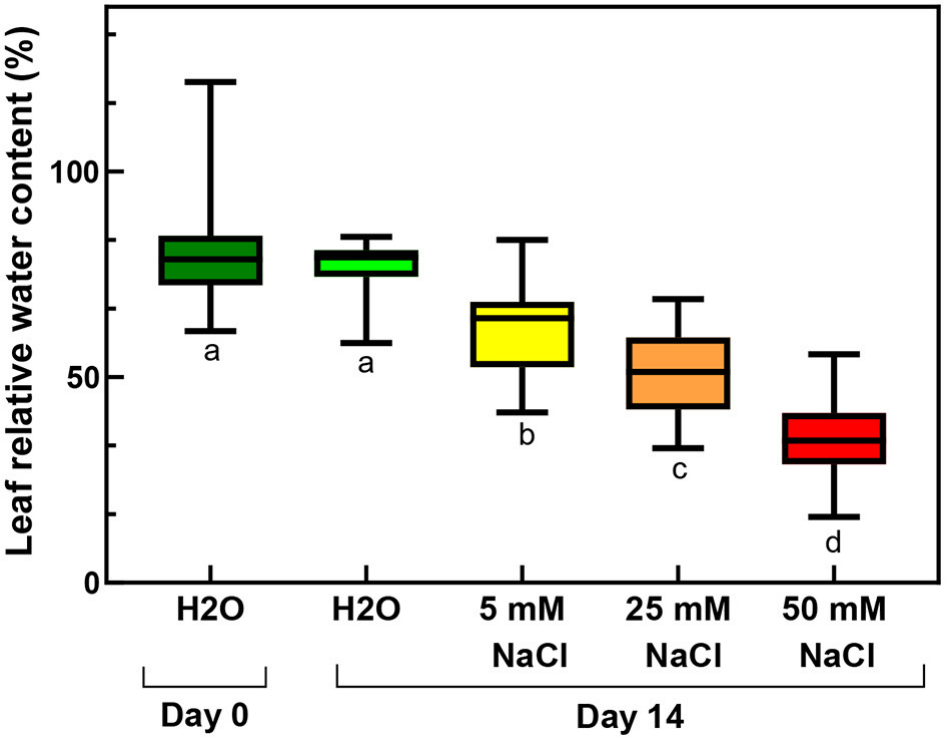
Relative water contents of third and fourth leaf pairs of freshly cut spearmint shoot before treatment (Day 0, distilled water, “H_2_O”), or after 2 weeks of treatment with 0 mM NaCl (Day 14, distilled water, “H_2_O”), 5, 25 and 50 mM NaCl (dissolved in distilled water, measured on Day 14). Different letters indicate statistically significant differences between the samples according to one-way ANOVA and Tukey-Kramer multiple comparisons test (*P* < 0.05) (*n* = 24–30).

**Figure 3:**
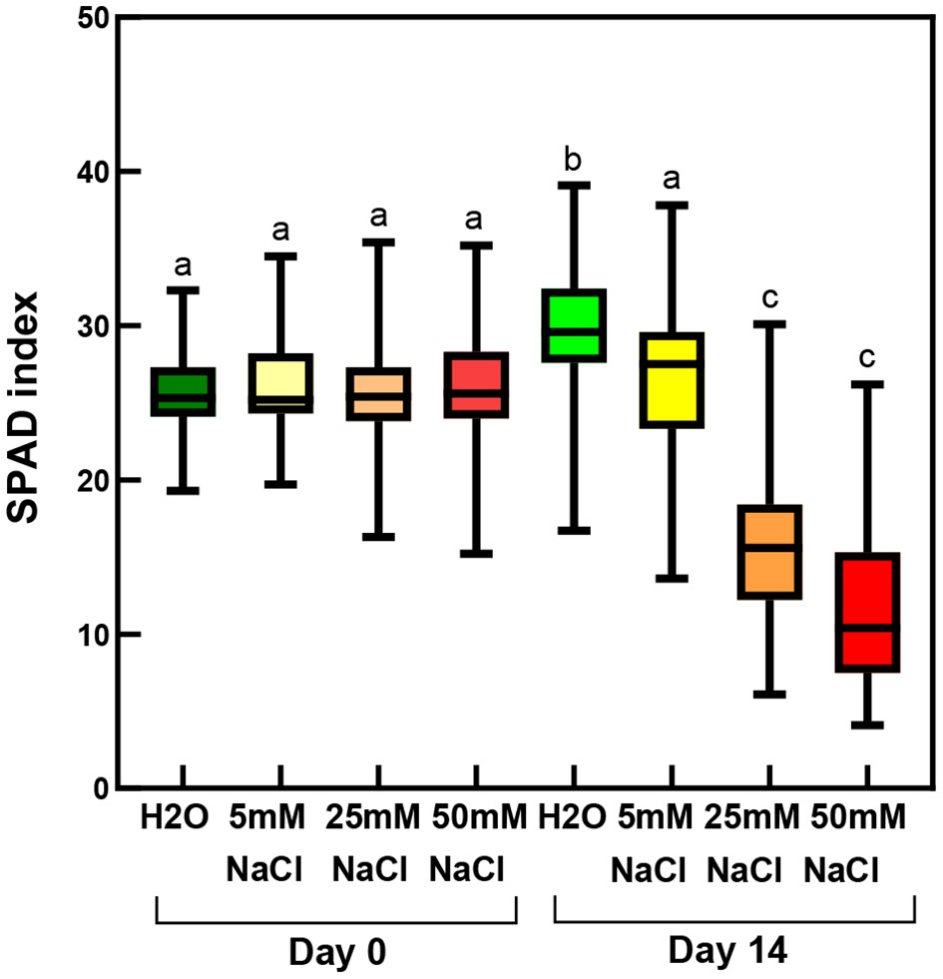
SPAD index of third and fourth leaf pairs of freshly cut spearmint shoots before treatment (“Day 0”), or after 2 weeks of treatment (“Day 14”) with 0 mM NaCl (H_2_O, distilled water), 5, 25, and 50 mM NaCl (dissolved in distilled water). Different letters indicate statistically significant differences between the samples according to Kruskal-Wallis non-parametric ANOVA followed by Dunn's multiple comparisons *post hoc* test (*P* < 0.05) (*n* = 120–147).

At 50 mM NaCl concentrations, the leaves started to show chlorotic symptoms, and the leaf margins or sometimes also the entire leaves became dry and brownish (Figure 1B). The RWC values of the leaves significantly and gradually decreased with the applied salt stress treatments of increasing severity (Figure 2; Supplementary Table 1), and their SPAD index values reflecting their relative chlorophyll contents also significantly decreased (Figure 3; Supplementary Table 2). The data showed that after 2 weeks of treatment, the control shoots (immersed into distilled water) had a significant increase in the chlorophyll contents of their third and fourth leaf pairs, which indicated that chlorophyll biosynthesis and accumulation was still active in them. Therefore, when compared with the control plants after 2 weeks, the leaves of the plants treated with all salt concentrations had a lower chlorophyll content, with drastically low values measured in the case of the plants treated with 50 mM NaCl (Figure 3).

**Figure 4:**
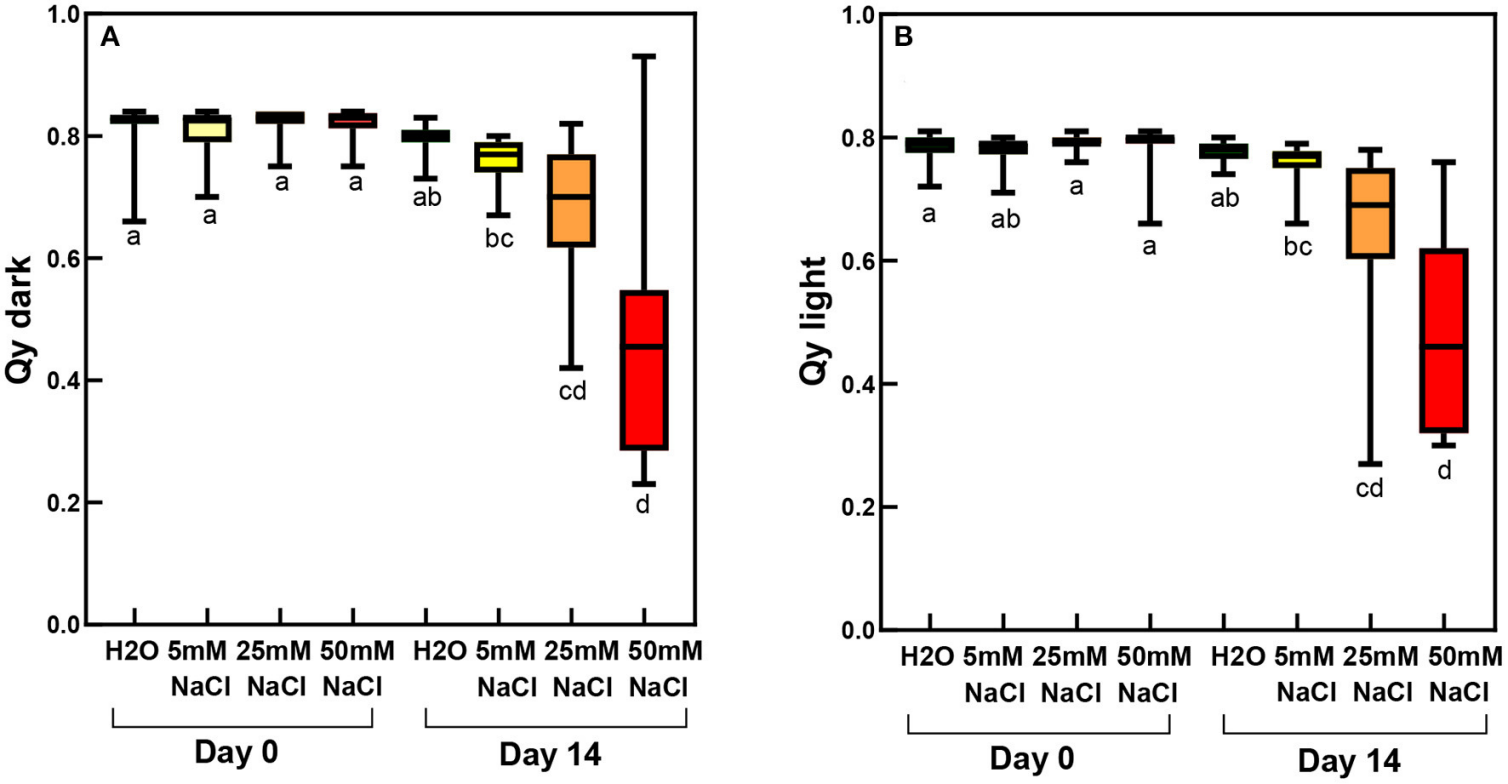
Actual (Qy light) or maximal (Qy dark) quantum efficiency of photosystem II in third and fourth leaf pairs of spearmint shoots before treatment (“Day 0”), or after 2 weeks of treatment (“Day 14”) with 0 mM NaCl (H_2_O, distilled water), 5 mM, 25 mM and 50 mM NaCl (dissolved in distilled water). **(A)** Qy dark values recorded after 20 min dark adaptation. **(B)** Qy light measured in light-adapted plants. Different letters indicate statistically significant differences between the samples according to Kruskal-Wallis non-parametric ANOVA followed by Dunn's multiple comparisons test (*P* < 0.05) (Qy dark, *n* = 24–36; Qy light, *n* = 23–33).

In order to check the activity of the photosynthetic apparatus, chlorophyll fast fluorescence induction parameters were recorded (Figure 4, Supplementary Table 2). The Qy dark values characterizing the maximal quantum efficiency of photosystem II were determined after 20 min of dark adaptation of the plants, while the actual quantum efficiency values (Qy light) were measured in light-adapted samples (Qy light). The higher the Qy value, the higher the PSII-related photosynthetic activity. After 2 weeks of treatment, both Qy values significantly and gradually decreased in the samples treated with increasing salt concentration, especially at 50 mM of salt concentration.

**Figure 5:**
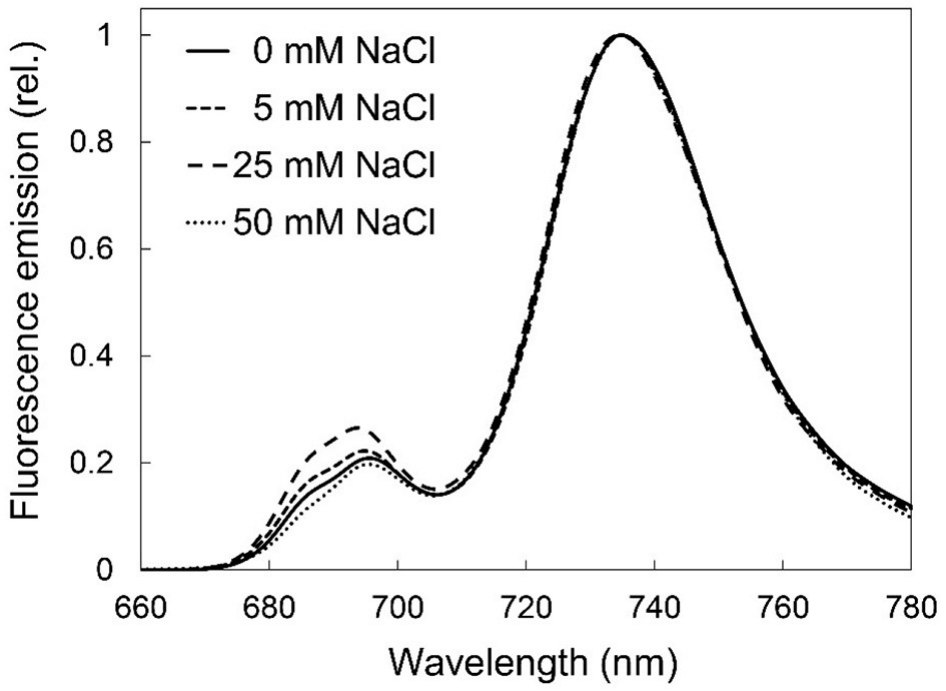
77K fluorescence spectra of third and fourth leaf pairs of freshly cut spearmint shoots treated with 0 mM (control, distilled water), 5, 25, and 50 mM NaCl in distilled water for 2 weeks. Figures show average spectra calculated after normalization (three independent experiments, *n* = 5–11). Excitation wavelength: 440 nm.

In order to characterize the organization of the chlorophyll-protein complexes of the photosynthetic apparatus, 77 K fluorescence emission spectra of the treated leaves were recorded, normalized, and averaged for each treatment (Figure 5). The relative fluorescence intensity of the emission bands of chlorophyll-protein complexes at 685 and 695 nm (which represented the chlorophyll-protein complexes CP43 and CP47 of photosystem II, PSII) showed slight but not important or specific differences among the treatments.

**Figure 6:**
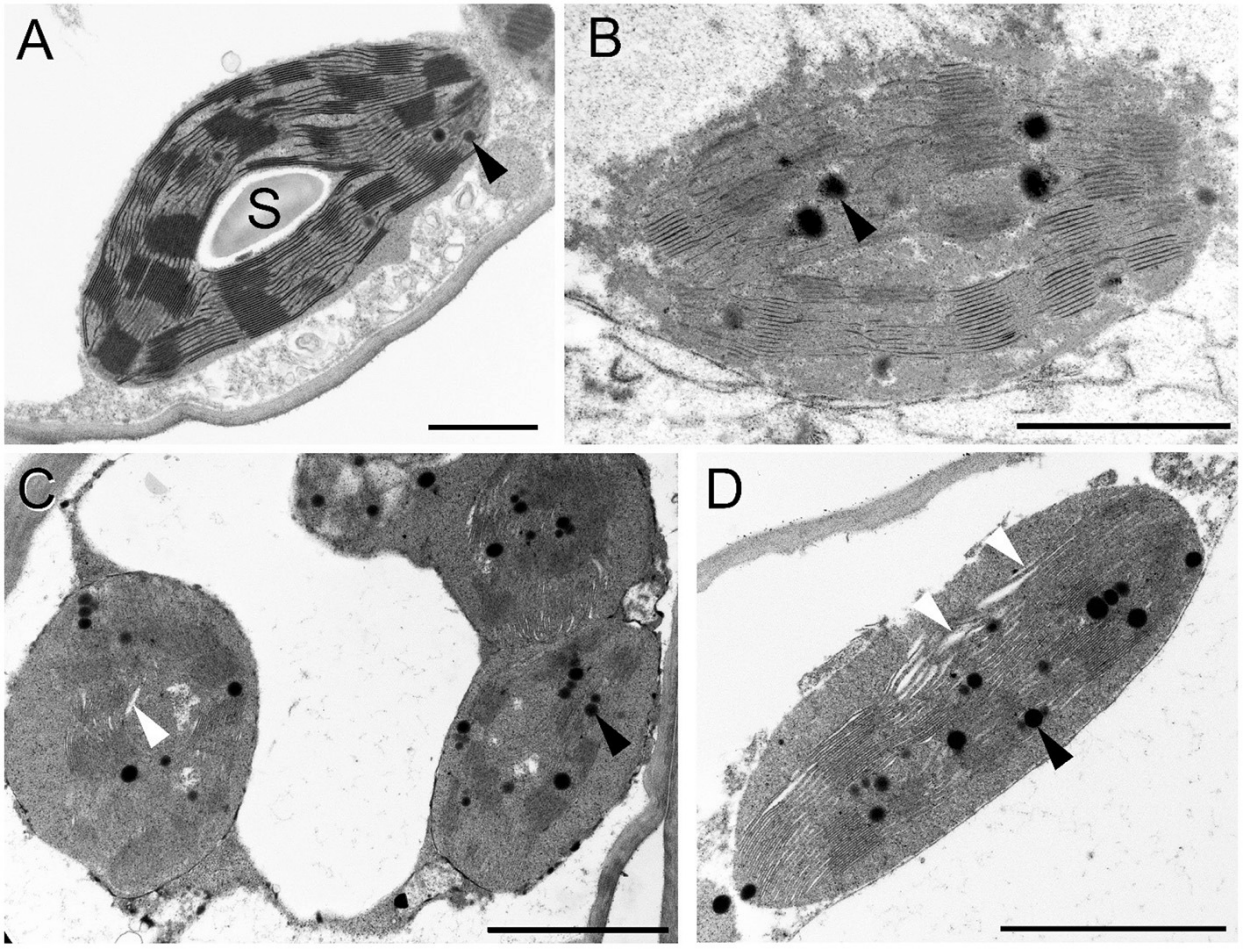
Transmission electron micrographs of chloroplasts in the fourth leaf pair of freshly cut spearmint shoots treated with 0 mM (control, distilled water) **(A)** and 50 mM NaCl in distilled water **(B–D)** for 2 weeks. B: brown, dry part of a leaf; **(C,D)** green, wilted region of the same leaf as in **(B)**. S: starch; black arrowhead: plastoglobule; white arrowhead: swollen intrathylakoidal space. Scale bar: 1 μm.

The ultrastructure of chloroplasts was analyzed using TEM. The control (0 mM NaCl, distilled water-treated) (Figure 6A), the 5 mM, and 25 mM NaCl-treated samples showed normal chloroplasts with grana, stroma thylakoids, and large starch grains after 2 weeks of treatment. For the 50 mM, NaCl treated samples we have separately sampled pale greenish and somewhat wilted leaf regions and fully brownish, dry, or drying leaf margin areas. Starch completely disappeared from the chloroplasts of these leaf regions (Figures 6B–D). Surprisingly, in the brownish and almost fully dry leaf regions, the chloroplasts and their thylakoid system, especially the grana could be still distinguished, although cellular integrity was already lost, and the chloroplast envelope was also hardly discernible (Figure 6B). In the green leaf region, the cells were plasmolyzed (Figure 6C), and slight swelling of the lumen of the stroma thylakoids and rarely of the granum end membranes was observed in these chloroplasts (Figures 6C,D, white arrowheads). The number of plastoglobuli slightly increased in the plants treated with 50 mM NaCl for 2 weeks.

After observing important changes in vegetative reproduction and activity and structure of the photosynthetic apparatus during the applied salt stress, we wanted to investigate the effect of the treatment on the essential oil composition of spearmint. Therefore, we determined the essential oil composition after SPME using GC/MS of plants treated for 2 weeks with the various solutions (Table 1). Monoterpenes were dominating in the essential oil (67–73%), with sesquiterpenes accounting for less than one-fourth of the compounds (23–31%), and partially volatile diterpenes representing a minor fraction (1–3%). Carvone was the major compound of the spearmint leaves (62–65%), followed by limonene (11–13%), germacrene D (7–9%), and β-caryophyllene (5–6%). No significant differences were observed in the individual components between the control (H_2_O, 0 mM NaCl) and the treated plants, except for γ-cadinene which is a minor component present in < 0.5% in the essential oil.

**Table 1:** Percentage composition of the essential oils produced by the third, fourth, and fifth leaf pairs of freshly cut spearmint shoots treated with 0 mM NaCl (control, distilled water, “H_2_O”), 5, 25, and 50 mM NaCl for 2 weeks at room temperature and ambient light conditions.

**Compounds**	**Retention index**	**Percentage ratio of the compounds (%)**			
		**H_2_O (*n* = 5)**	**5 mM NaCl (*n* = 5)**	**25 mM NaCl (*n* = 5)**	**50 mM NaCl (*n* = 5)**
α-Pinene	923	Tr.	Tr.	0.2 ± 0.1a	0.2 ± 0.1a
β-Pinene	968	Tr.	Tr.	0.2 ± 0.1a	0.2 ± 0.1a
β-Myrcene	982	0.4 ± 0.2a	0.6 ± 0.0a	0.8 ± 0.2a	0.8 ± 0.1a
Limonene	1,023	10.6 ± 1.9a	10.6 ± 0.8a	12.2 ± 1.9a	12.9 ± 1.3a
β-Ocimene (E)	1,027	0.6 ± 0.2a	0.7 ± 0.0a	1.0 ± 0.1a	0.7 ± 0.2a
Carvone	1,247	63.3 ± 2.6a	61.9 ± 3.3a	65.0 ± 5.7a	64.6 ± 6.0a
Piperitone	1,258	0.5 ± 0.0a	0.3 ± 0.1a	0.4 ± 0.1a	0.3 ± 0.1a
Carvyl acetate	1,356	Tr.	Tr.	0.3 ± 0.2a	0.7 ± 0.3a
β-Bourbonene	1,383	1.0 ± 0.1a	1.2 ± 0.1a	0.9 ± 0.1a	0.8 ± 0.2a
α-Gurjunene	1,406	Tr.	Tr.	Tr.	Tr.
β-Caryophyllene	1,424	5.9 ± 0.6a	6.4 ± 0.7a	4.7 ± 1.0a	4.8 ± 1.2a
Epi-bicyclosesquiphellandrene	1,447	2.1 ± 0.4a	2.0 ± 0.3a	1.4 ± 0.4a	1.0 ± 0.3a
α-Humulene	1,459	0.8 ± 0.1a	0.9 ± 0.1a	0.7 ± 0.2a	0.7 ± 0.2a
γ-Gurjunene	1,465	2.0 ± 0.3a	2.2 ± 0.2a	1.6 ± 0.4a	1.5 ± 0.4a
Germacrene D	1,482	8.4 ± 1.6a	8.9 ± 1.3a	7.1 ± 2.1a	6.9 ± 2.0a
γ-Cadinene	1,513	0.4 ± 0.1ab	0.5 ± 0.0a	0.2 ± 0.1ab	0.1 ± 0.1b
β-Cadinene	1,519	0.5 ± 0.1a	0.5 ± 0.1a	0.4 ± 0.1a	0.4 ± 0.1a
Cis-calamenene	1,525	0.3 ± 0.2a	0.4 ± 0.2a	0.3 ± 0.2a	0.6 ± 0.3a
α-Muurolene	1,538	0.3 ± 0.1a	0.4 ± 0.1a	0.2 ± 0.1a	0.2 ± 0.1a
δ-Cadinol	1,620	0.5 ± 0.2a	0.6 ± 0.1a	0.4 ± 0.2a	0.5 ± 0.2a
α-Cadinol	1,660	0.3 ± 0.2a	0.4 ± 0.1a	0.2 ± 0.2a	0.2 ± 0.1a
Labdane derivative	2,114	1.1 ± 0.7a	0.6 ± 0.3a	0.4 ± 0.3a	0.5 ± 0.4a
Monoterpenes	73.4	67.1	67.1	68.0	
Sesquiterpenes	23.0	30.7	30.8	29.7	
Diterpenes	2.6	1.4	0.9	1.3	

*The experiments were carried out in five independent biological replicates as indicated, and for each measurement pooled samples (0,5–1 g plant material) were used. The mean values and standard errors of the mean are indicated in the Table. Different letters indicate significant differences according to Kruskal-Wallis non-parametric ANOVA followed by Dunn's multiple comparisons post hoc test (P < 0.05). “Tr.” refers to compounds detected in very low amounts (≤0.1%)*.

### The Effect of High Concentration Salt Stress Treatments on Spearmint

Considering the striking results which showed that adventitious root formation is already inhibited at 50 mM NaCl treatments, we wanted to study how the presence of roots influenced salt stress in spearmint. Also, in view of the observed slight swelling of the intrathylakoidal space of the chloroplasts of leaves of plants treated with 50 mM NaCl for 2 weeks, we wanted to investigate whether the specific ionic or the osmotic components of salt stress could be related to the ultrastructural and functional alterations of the photosynthetic apparatus observed under salt stress. We also wanted to check whether strong salt stress influenced the essential oil composition of mint or not. Therefore, in the next experimental setup, we used a high concentration of NaCl (150 mM) and applied it to freshly cut (“rootless”) and rooted shoots for 1 week under ambient conditions. We used distilled water as control and an isosmotic PEG solution which had the same osmolarity, i.e., 283 mOsm as the 150 mM NaCl solution in order to model the osmotic component of salt stress.

**Figure 7:**
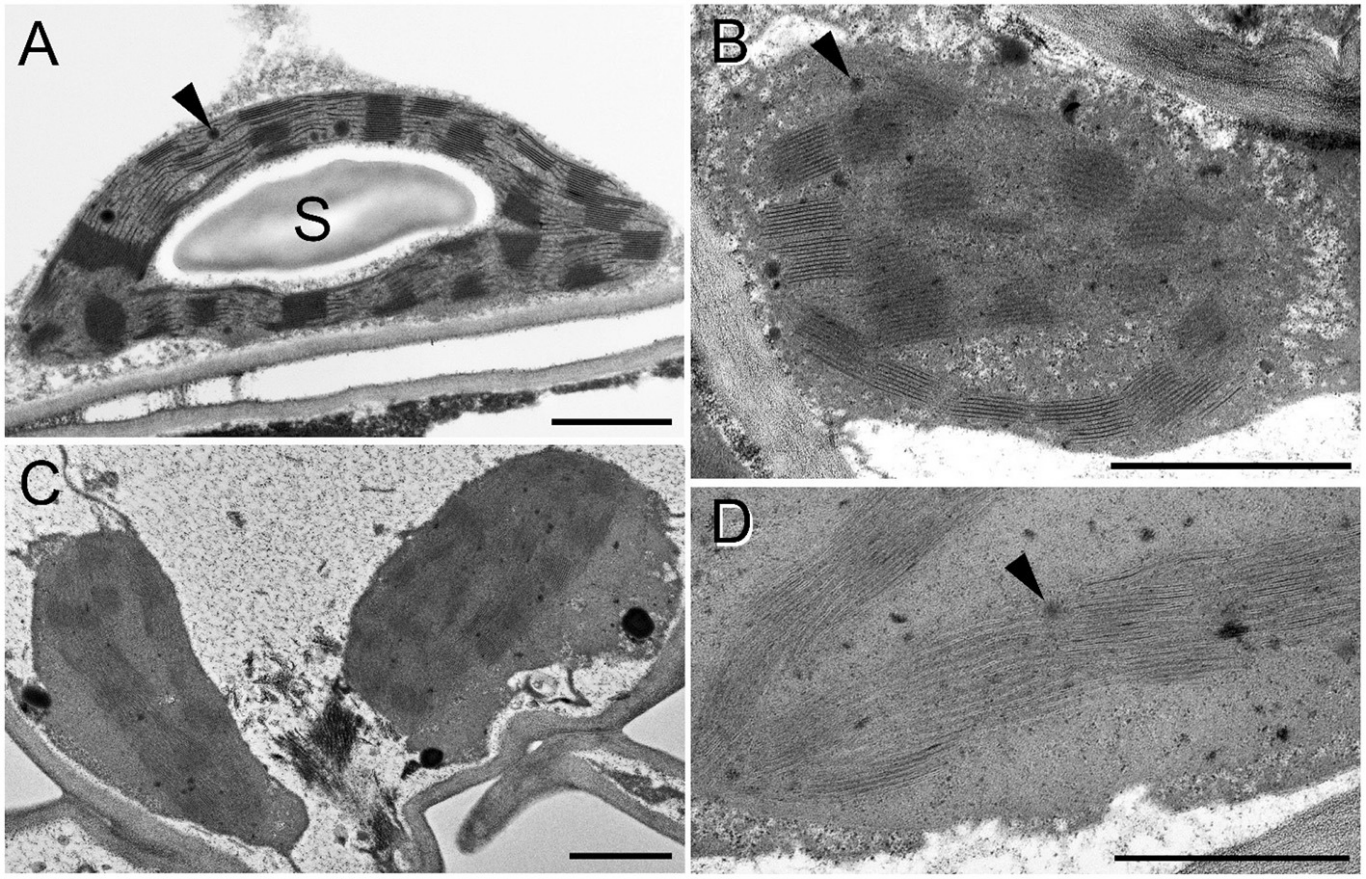
Transmission electron micrographs of chloroplasts in the fourth leaf pair of freshly cut, i.e., rootless spearmint shoots treated with 0 mM NaCl (control, distilled water), 150 mM NaCl (in distilled water), and polyethylene glycol (PEG-6000, in distilled water, with equal osmolarity to the 150 mM NaCl solution) for 1 week. **(A)** 0 mM NaCl (control, distilled water); **(B)** PEG-6000 treatment; **(C,D)** treatment with 150 mM NaCl. S: starch; black arrowhead: plastoglobule. Scale bar: 1 μm.

Freshly cut, rootless shoots and their leaves were fully dried and had brownish color after 1 week of treatment both in 150 mM NaCl and in the isosmotic PEG solution (compare Figures 1C,D while control plants (immersed in distilled water) were green and had normal chloroplasts (Figure 7A). TEM fixation showed cellular disintegration and chloroplast disorganization (unclear appearance of the envelope membranes) in these rootless plants both when treated with 150 mM NaCl and isosmotic PEG for 1 week (Figures 7B–D). However, grana and plastid inner membranes were relatively and surprisingly well retained in both cases.

**Figure 8:**
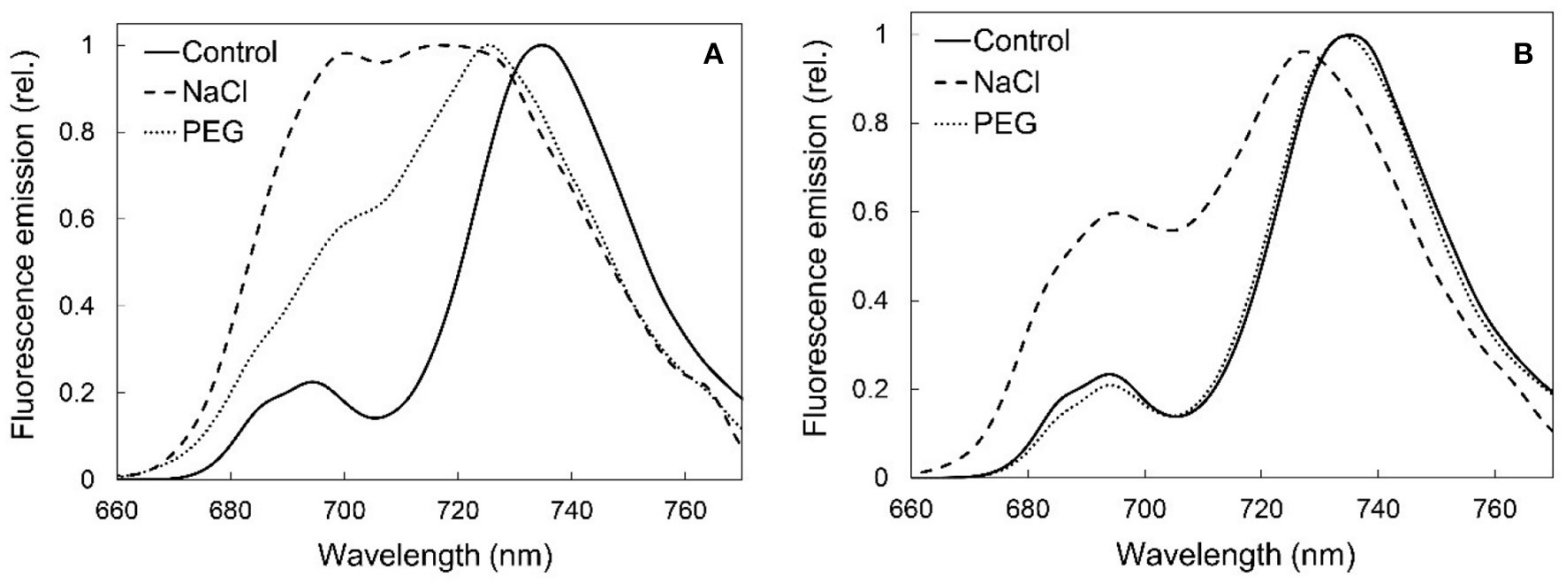
77K fluorescence spectra of leaf pieces from third and fourth leaf pairs of spearmint plants treated with 0 mM (control, distilled water), 150 mM NaCl (in distilled water), or isosmotic polyethylene glycol (PEG-6000, in distilled water, with equal osmolarity to the 150 mM NaCl solution) for 1 week. **(A)** freshly cut, i.e., rootless plants; **(B)** rooted plants. Figures show average spectra calculated after normalization (*n* = 3–6). Excitation wavelength: 440 nm.

The fluorescence emission spectra of the native chlorophyll-protein complexes of freshly cut or rooted plants showed important alterations in the fluorescence of the pigment forms under salt treatment (Figure 8). Such reorganizations were also observed in the rootless plants treated with PEG (Figure 8A), but in the case of the rooted plants, osmotic stress, i.e., isosmotic PEG treatment, did not induce changes in the fluorescence emission spectra of the chlorophyll-protein complexes of the photosynthetic apparatus when compared with the control (Figure 8B). This is in accordance with the phenotype of these plants which only slightly changed under the treatment (compare Figures 1E,F).

**Figure 9:**
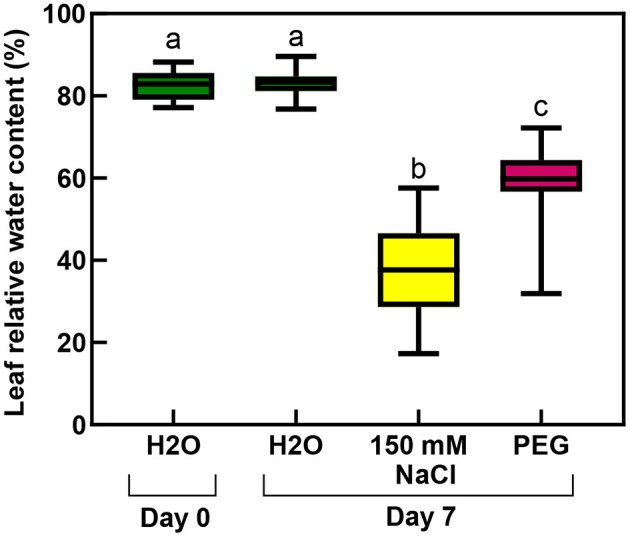
SPAD index values of third and fourth leaf pairs of rooted spearmint treated with 0 mM NaCl (“H_2_O,” control, distilled water), 150 mM NaCl, and isosmotic polyethylene glycol (PEG-6000, in distilled water, with equal osmolarity to the 150 mM NaCl solution) after 1 week of treatment (“Day 7”). For comparison, the values measured before the 1-week-long treatment are also indicated (“Day 0”). Different letters indicate statistically significant differences between the samples according to Kruskal-Wallis non-parametric ANOVA followed by Dunn's multiple comparisons *post hoc* test (*P* < 0.05) (*n* = 80–93).

We performed acetonic extraction of the pigments, but using fluorescence spectroscopy of the diluted extracts, we did not observe pheophytin formation in the brown leaves. Chlorophyll *a* and *b* were detected in the extracts, thus, the brownish coloration was caused by other pigments than pheophytin *a* or *b*. Quantification of the pigments based on acetonic extracts and a fresh mass basis of the leaves was difficult due to the different extent of drying and RWC values of the various leaves or variously dried leaf regions, e.g., leaf margins were drying more rapidly than the middle region of the leaves. Therefore, we later used the SPAD index to characterize the relative chlorophyll content of the middle leaf regions (Figure 9). However, it has to be noted that in the case of the almost fully dry, fragile, brownish leaves of freshly cut, i.e., rootless shoots, SPAD and RWC measurements were not carried out.

No significant changes occurred in the leaf SPAD index values of control rooted plants during the 1-week-long treatment on distilled water (H_2_O), but significantly decreased SPAD index values showed a lower relative chlorophyll content in the osmotic stressed (PEG-treated) leaves, and the lowest chlorophyll content was observed in the 150 mM NaCl-treated plants (Figure 9; Supplementary Table 3).

**Figure 10:**
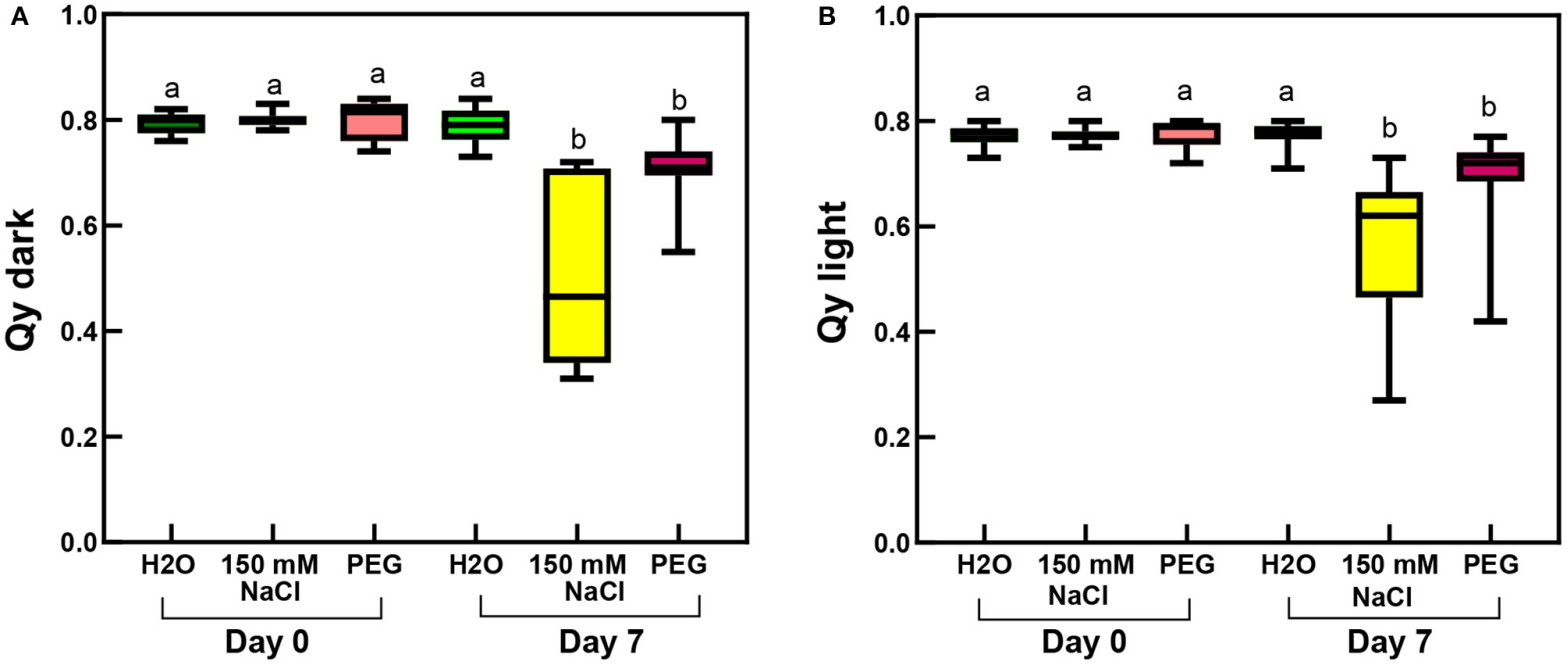
Qy parameters of third and fourth leaf pairs of rooted spearmint shoots before treatment (“Day 0”), or after 1 week of treatment (“Day 7”) with 0 mM NaCl (“H_2_O,” control, distilled water), 150 mM NaCl and isosmotic polyethylene glycol (PEG-6000, in distilled water, with equal osmolarity to the 150 mM NaCl solution). **(A)** Qy dark values recorded after 20 min dark adaptation. **(B)** Qy light measured in light-adapted plants. Different letters indicate statistically significant differences between the samples according to Kruskal-Wallis non-parametric ANOVA followed by Dunn's multiple comparisons *post hoc* test (*P* < 0.05) (Qy dark, *n* = 16–24; Qy light, *n* = 21–29).

Considering the photosynthetic activity in rooted plants, not surprisingly, the largest decrease in the maximal and the actual PSII quantum efficiencies (as measured by Qy parameters recorded in the dark-adapted and light-adapted states, respectively) was observed in the salt-stressed sample, and slight but also significant decrease occurred in the PEG-treated leaves (Figure 10; Supplementary Table 3).

**Figure 11:**
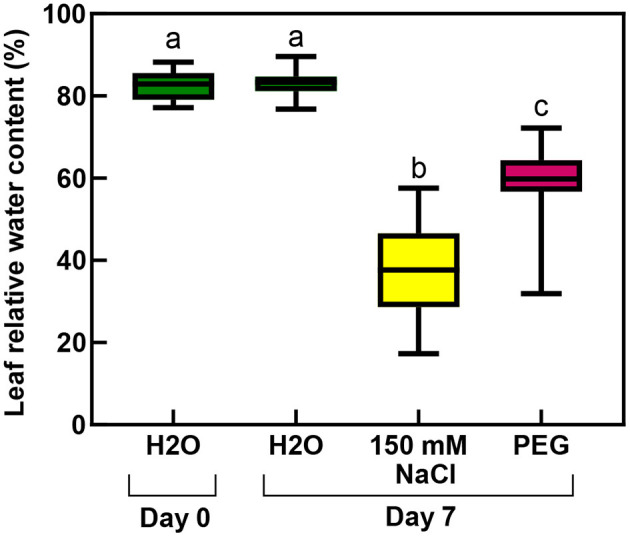
Relative water contents of third and fourth leaf pairs of rooted spearmint shoots before treatment (“Day 0”), or after 1 week of treatment (“Day 7”) with 0 mM NaCl (“H_2_O,” control, distilled water), 150 mM NaCl and isosmotic polyethylene glycol (PEG-6000, in distilled water, with equal osmolarity to the 150 mM NaCl solution). Different letters indicate statistically significant differences between the samples according to one-way ANOVA and Tukey-Kramer multiple comparison test (*P* < 0.05) (*n* = 18).

The RWC values of the rooted plants were 83% before the treatment, which significantly decreased during the salt stress to 37%, and a smaller but still significantly lower level (to 59%) by the osmotic stress (isosmotic PEG-treatment) (Figure 11; Supplementary Table 4).

**Figure 12:**
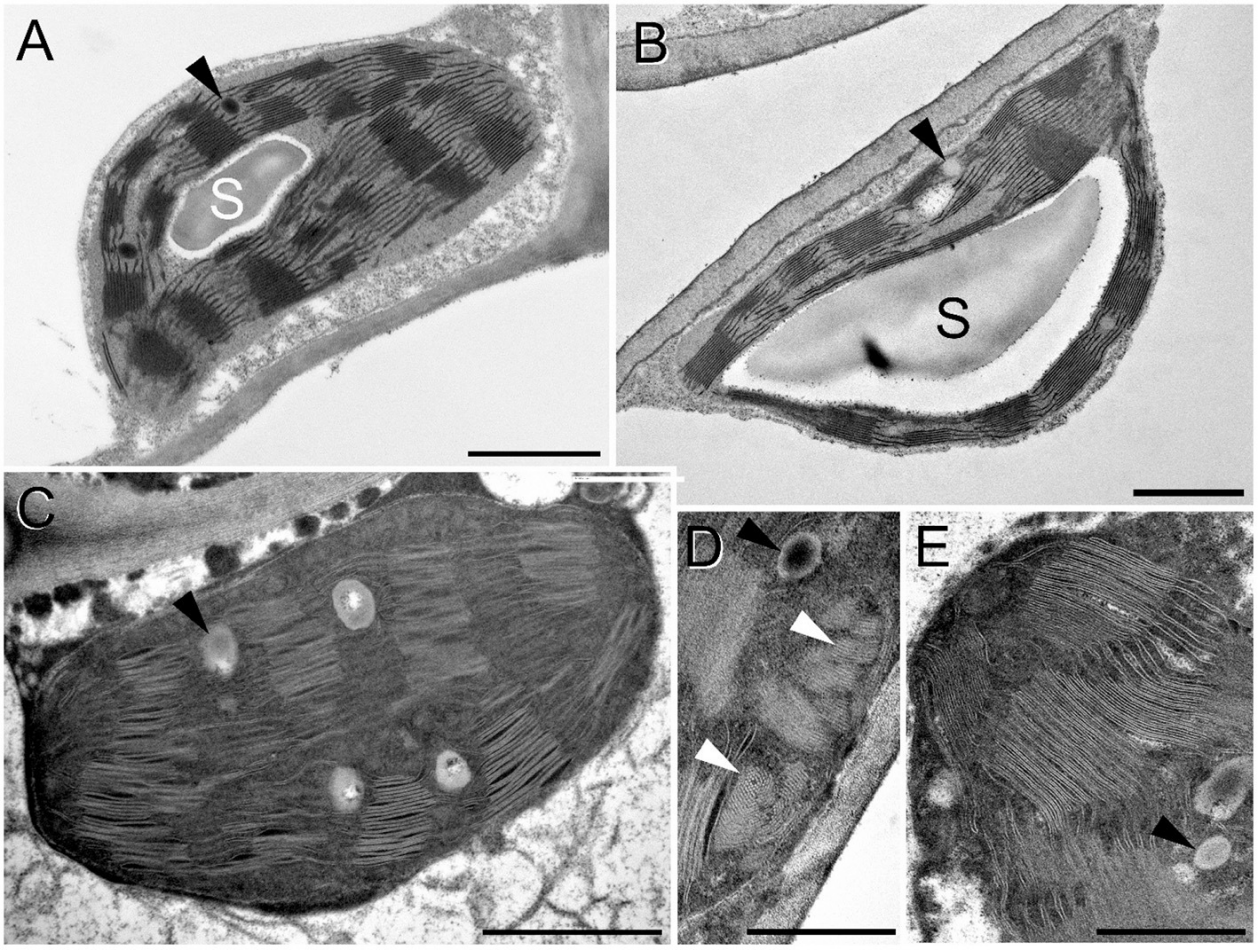
Transmission electron micrographs of chloroplasts in the fourth leaf pair of rooted spearmint shoots treated with 0 mM NaCl (control, distilled water), 150 mM NaCl (in distilled water), and isosmotic polyethylene glycol (PEG-6000, in distilled water, with equal osmolarity to the 150 mM NaCl solution) for 1 week. **(A)** control (H_2_O); **(B)** PEG; **(C–E)** 150 mM NaCl; C and D: from brown, dry part of the leaf; E: green, wilted region of the same leaf as in C and D. S: starch; black arrowhead: plastoglobule; white arrowhead: crystalline inclusion. Scale bar: 1 μm **(A–C)**, 0,5 μm **(D,E)**.

Considering chloroplast ultrastructure, rooted plants tolerated relatively well the osmotic stress, i.e., the osmotic component of the 150 mM NaCl stress which was applied using an isosmotic solution of PEG, as revealed by TEM analyses (Figure 12). Control chloroplasts (Figure 12A) looked similar to the plastids of rooted, PEG-treated leaves (Figure 12B). The only difference we observed was a change in the electron density of the plastoglobuli, wherein they were electron-dense in the control plants while they became electron-transparent in the PEG-treated leaves (Figure 12, black arrowheads). However, in spearmint plants treated with 150 mM NaCl for 1 week through the roots, cells were fully disorganized both in the brownish and greenish leaf regions and had similar plastids (Figures 12C–E). The chloroplast stroma became highly electron-dense, the size of plastoglobuli increased, and the thylakoid system became disorganized, grana lost their regular periodicity, and crystalline spotted electron-transparent inclusions appeared in the plastids (Figure 12D, white arrowheads).

The granum RD values determined based on the FFT analyses of the TEM images were 18.36 ± 0.14 nm (*n* = 170) and 18.90 ± 0.35 nm (*n* = 147), in control and PEG-treated samples, respectively. A T-test revealed no significant difference between the parameters observed in the two different samples. In the case of salt-stressed leaves, the RD values could not be precisely determined due to the fuzziness of the FFT images.

**Figure 13:**
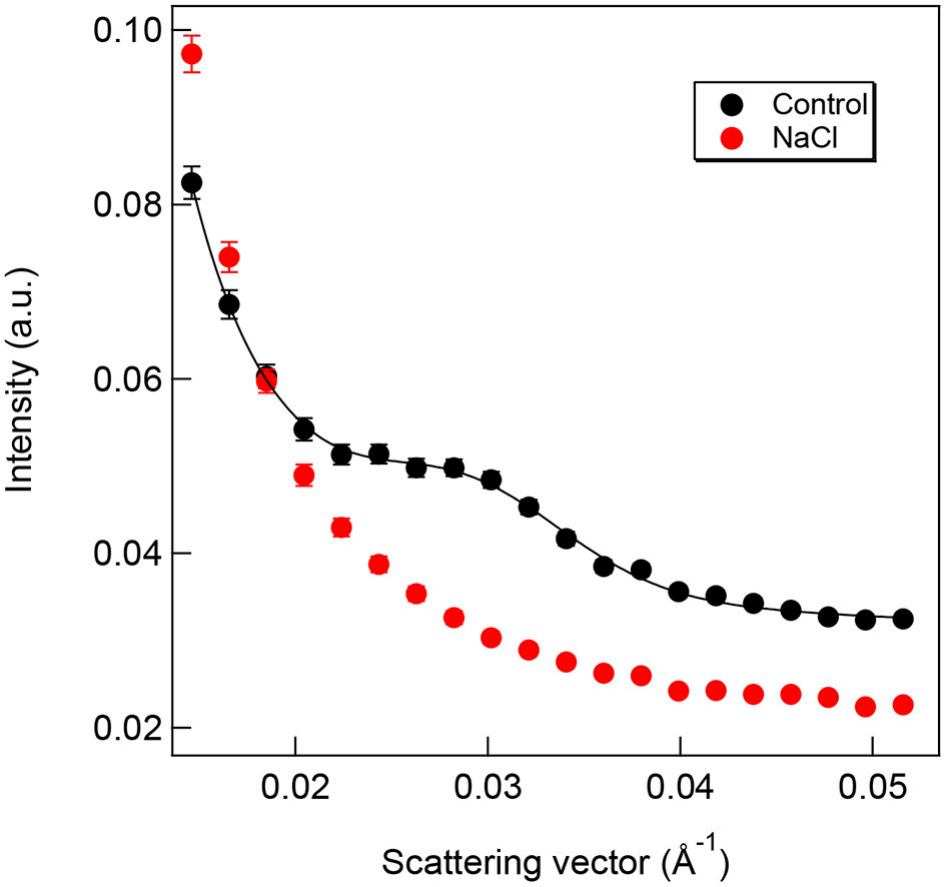
Typical radially averaged SANS curves of the upper part (first four leaf pairs and shoot tip) of whole intact rooted spearmint shoots treated for 1 week with 0 mM NaCl (control, distilled water; black curve) or 150 mM NaCl (in distilled water, red curve), black solid line is the fitted curve.

In order to investigate whether (i) it is possible to determine RD values of intact spearmint leaves *in vivo* by SANS, and (ii) whether the differences observed by TEM in the granum regularity of control and salt-stressed plants are not fixation artifacts, we carried out SANS measurements on rooted spearmint shoots treated with 0 mM and 150 mM NaCl. The presence of the Bragg peak related to the periodicity of granal thylakoid membranes at about 0.03 Å^−1^ on the SANS curve strongly depended on the periodic order of the thylakoid membranes. The rooted plants treated with 150 mM NaCl had disorganized granal thylakoid membranes and had no clear SANS signal, while the intact, rooted control plants contained periodic multilamellar granal thylakoid membranes and had a nice Bragg peak (Figure 13). The averaged RD value was 212 ± 7 Å (*n* = 3), which was somewhat larger than the RD values obtained by TEM. The origin of this difference was not fully understood, although it most likely originated from fixation artifacts during the preparation of samples for TEM (Ünnep et al., 2014). Hence, it can be concluded that the observed irregular ultrastructure and disrupted regularity of granal thylakoid membranes revealed by TEM (Figure 12) were consistent with SANS data obtained in intact leaves (Figure 13).

Finally, we wanted to study the effect of the salt and osmotic stress treatments on the essential oil composition of the plants. No significant changes were observed in the essential oil composition of the salt and PEG-treated rootless plants when compared with the control plants (immersed in distilled water for 1 week) except for a minor compound, β-bourbonene, the relative amount of which slightly but significantly increased under salt stress (Table 2). Similar results were found, when we compared the essential oil composition of the rooted plants, in which another minor compound, α-Cadinol, showed an almost minimal, but statistically significant increase in the PEG-treated plants (Table 2). Our data showed that 1-week-long salt and osmotic stress do not significantly alter the aromatic properties of the third, fourth and fifth leaf pairs of spearmint shoots.

**Table 2:** Percentage composition of the essential oils produced by the third, fourth and fifth leaf pairs of rooted or freshly cut (“rootless”) spearmint shoots treated with 0 mM NaCl (control, distilled water, “H_2_O”), 150 mM NaCl, or isosmotic PEG-6000 solution (PEG-6000, in distilled water, with equal osmolarity to the 150 mM NaCl solution) for 1 week at room temperature and ambient light conditions.

**Compounds**	**Retention index**	**Percentage ratio of the compounds (%)**					
		**H_2_O rooted (*n* = 5)**	**150 mM NaCl rooted (*n* = 5)**	**PEG rooted (*n* = 5)**	**H_2_O rootless (*n* = 3)**	**150 mM NaCl rootless (*n* = 3)**	**PEG rootless (*n* = 3)**
α-Pinene	923	0.4 ± 0.0a	0.4 ± 0.1a	0.3 ± 0.0a	0.2 ± 0.2^*^	0.2 ± 0.1^*^	0.2 ± 0.1^*^
β-Pinene	968	0.4 ± 0.0a	0.5 ± 0.1a	0.4 ± 0.0a	0.2 ± 0.1^*^	0.3 ± 0.1^*^	0.2 ± 0.1^*^
β-Myrcene	982	1.3 ± 0.1a	1.3 ± 0.2a	1.3 ± 0.1a	1.0 ± 0.2^*^	1.1 ± 0.2^*^	0.9 ± 0.0^*^
Limonene	1,023	14.0 ± 0.3a	13.1 ± 0.9a	12.7 ± 1.2a	19.7 ± 4.3^*^	21 ± 1.2^*^	16.5 ± 1.0^*^
β-Ocimene (E)	1,027	1.5 ± 0.1a	1.5 ± 0.2a	1.0 ± 0.3a	1.1 ± 0.1^*^	1.0 ± 0.0^*^	0.9 ± 0.1^*^
Borneol	1,167	0.2 ± 0.1a	0.2 ± 0.1a	0.3 ± 0.0a	Tr.	Tr.	Tr.
Dihydrocarvon	1,200	0.7 ± 0.4a	0.4 ± 0.1a	0.5 ± 0.1a	Tr.	Tr.	Tr.
Carvone	1,247	46.6 ± 3.0a	43.3 ± 1.3a	44.0 ± 1.1a	59.1 ± 3.2^*^	51.2 ± 5.3^*^	62.6 ± 4.3^*^
Piperitone	1,258	0.5 ± 0.1a	0.6 ± 0.1a	0.5 ± 0.1a	0.4 ± 0.2^*^	0.4 ± 0.2^*^	0.4 ± 0.2^*^
α-Copaene	1,378	0.2 ± 0.1a	0.2 ± 0.1a	0.3 ± 0.0a	Tr.	Tr.	Tr.
β-Bourbonene	1,383	1.9 ± 0.5a	2.2 ± 0.4a	2.0 ± 0.4a	1.3 ± 0.3^*^	2.2 ± 0.1^**^	1.3 ± 0.2^*^
α-Gurjunene	1,406	0.4 ± 0.0a	0.5 ± 0.0a	0.5 ± 0.1a	Tr.	Tr.	Tr.
β-Caryophyllene	1,424	7.8 ± 0.5a	8.6 ± 0.4a	8.7 ± 0.5a	5.1 ± 0.3^*^	6.7 ± 0.8^*^	4.7 ± 0.8^*^
Amorphene	1,429	0.3 ± 0.1a	0.4 ± 0.0a	0.3 ± 0.0a	Tr.	Tr.	Tr.
Epi-bicyclosesquiphellandrene	1,447	2.9 ± 0.1a	3.0 ± 0.2a	3.4 ± 0.3a	1.4 ± 0.2^*^	1.1 ± 0.2^*^	1.3 ± 0.3^*^
α-Humulene	1,459	1.2 ± 0.1a	1.3 ± 0.1a	1.1 ± 0.3a	0.7 ± 0.1^*^	0.8 ± 0.1^*^	0.6 ± 0.1^*^
γ-Gurjunene	1,465	2.8 ± 0.2a	3.2 ± 0.1a	3.7 ± 0.5a	1.7 ± 0.3^*^	2.1 ± 0.3^*^	1.7 ± 0.2^*^
Germacrene D	1,482	10.1 ± 0.5a	12.0 ± 0.9a	11.9 ± 0.7a	6.1 ± 0.3^*^	6.8 ± 1.5^*^	5.8 ± 0.9^*^
γ-Cadinene	1,513	0.4 ± 0.0a	0.5 ± 0.0a	0.4 ± 0.1a	Tr.	Tr.	Tr.
β-Cadinene	1,519	0.3 ± 0.1a	0.5 ± 0.0a	0.4 ± 0.1a	0.8 ± 0.3^*^	1.4 ± 0.2^*^	0.8 ± 0.2^*^
cis-Calamenene	1,525	0.8 ± 0.2a	1.0 ± 0.1a	1.1 ± 0.3a	Tr.	Tr.	Tr.
α-Muurolene	1,538	0.3 ± 0.1a	0.6 ± 0.0a	0.6 ± 0.1a	Tr.	Tr.	Tr.
Caryophyllene-oxide	1,588	0.2 ± 0.1a	0.2 ± 0.1a	0.2 ± 0.1a	Tr.	Tr.	Tr.
δ-Cadinol	1,620	0.8 ± 0.1a	1.0 ± 0.1a	1.0 ± 0.0a	0.3 ± 0.2^*^	0.8 ± 0.1^*^	0.7 ± 0.3^*^
τ-Cadinol	1,647	0.2 ± 0.1a	0.4 ± 0.1a	0.4 ± 0.0a	Tr.	Tr.	Tr.
α-Cadinol	1,660	0.5 ± 0.0a	0.6 ± 0.1ab	0.6 ± 0.0b	0.3 ± 0.1^*^	0.7 ± 0.1^*^	0.3 ± 0.3^*^
Labdane derivative	2,114	1.6 ± 0.4a	1.4 ± 0.5a	1.4 ± 0.2a	Tr.	Tr.	Tr.
Monoterpenes		74.1	64.0	73.0	84.8	66.1	75.4
Sesquiterpenes		21.7	33.2	25.5	14.2	28.9	22.1
Diterpenes		3.4	1.2	1.2	0.0	4.9	1.8

The experiments were carried out in five or three independent biological repetitions as indicated, and for each measurement pooled samples (0,5–1 g plant material) were used. The mean values and standard errors of the mean are indicated in the Table. No significant differences were found among the different treatments according to one-way-ANOVA (P < 0.05) as indicated by similar letters (a) and asterisks (^*^). Different letters indicate significant differences according to Kruskal-Wallis non-parametric ANOVA followed by Dunn's multiple comparisons post hoc test (P < 0.05). Different numbers of asterisks indicate significant differences according to one-way-ANOVA followed by Tukey-Kramer multiple comparisons post hoc test (P < 0.05). “Tr.” refers to compounds detected in very low amounts (≤0.1%).

## Discussion

Salinity is complex stress, which under natural conditions is often combined with high temperature and drought stress resulting in increased salt concentrations in the soil water. According to recent estimations, salinity and sodicity affect one billion hectares worldwide, which represent 10% of the total arable lands on Earth. As a consequence, the annual economic losses associated with salinity stress can be as much as 27 billion USD (Shahid et al., 2018). Agriculture already faces challenges to produce enough staple food and feed crops to nourish the growing human population. Therefore, the increasing demand for the production of herbs and aromatic plants may not be met. In this respect, a better understanding of the physiological, cellular, and molecular mechanisms of spearmint under salinity stress may yield useful information for tolerance screening and may also provide a theoretical basis for further developments, potential future breeding, cultivation, and utilization of spearmint in saline areas.

When looking at literature data before starting these experiments in January 2019, we found several reports on salinity stress in peppermint (Li et al., 2015) or other mint species (Aziz et al., 2008; Oueslati et al., 2010; Yu et al., 2015), but almost no data were available about spearmint (Chrysargyris et al., 2019). In addition, literature data primarily focused on plant growth parameters (such as biomass), essential oil yield and composition, as well as the sometimes antioxidant response of the mint species under salt stress.

Therefore, one of the aims of this study was to better understand whether spearmint tolerates salinity stress and can be cultivated on saline soils. The results of the first experiment indicated that 50 mM NaCl stress inhibited adventitious root formation (Figure 1B), which under normal circumstances is an important means of vegetative reproduction, propagation, and regeneration in mint species (Li et al., 1999; Gomes et al., 2015; Salehi et al., 2018). Therefore, large-scale industrial cultivation of spearmint in soils with high salinity may be limited.

The second experiment outlined the obvious, but still highly important role of roots in protecting plants against salinity and especially osmotic stress. In freshly cut, i.e., rootless shoots, the drying induced by osmotic stress (PEG treatment) and salt stress (150 mM NaCl) was faster, ultrastructural damage were more pronounced (compare Figures 7, 12), and the disorganization of the chlorophyll-protein complexes of the photosynthetic apparatus proceeded faster than in rooted plants (Figure 8). Indeed, our data outlined that rooted plants better tolerate osmotic stress alone than the combined specific ionic and osmotic stress induced by salt treatment.

Concerning the potential aromatic, culinary, and medicinal uses, in this work, we compared the effect of salt and osmotic stress on the essential oil composition (Tables 1, 2). Essential oils of *Lamiaceae* plants are produced by their capitate and peltate glandular hairs, which are carbon heterotrophic and depend on the metabolites produced by the adjoining photosynthetic cells for a continuous supply of carbon precursors (Aziz et al., 2008; Böszörményi et al., 2020). Stress-induced disruption of normal metabolism, e.g., photosynthesis and secondary metabolite production, often results in reduced growth of plants (Aziz et al., 2008; Kasrati et al., 2014), altered leaf or glandular hair differentiation, and could be expected to interfere with essential oil biosynthesis resulting in lower essential oil quantity and altered composition (Aziz et al., 2008; Kasrati et al., 2014; Sarmoum et al., 2019). Our data showed a typical spearmint essential oil profile characterized by the major presence of carvone (between 44 and 68%) as described by other studies (Kokkini et al., 1995; Cirlini et al., 2016; Chrysargyris et al., 2017, 2019). In the present study, limonene represented 11–17% of the essential oil, but mentone was not detected. Other important constituents of the samples were epi-bicyclosesquiphellandrene, germacrene D and β-caryophyllene (Tables 1, 2). Variation in the composition of essential oils can be attributed to different factors such as temperature, humidity, climate, soil type as well as the cultivar studied, and in this respect spearmint essential oil profiles greatly vary in the literature (Sarmoum et al., 2019; Zekri et al., 2019), and also showed some variation within our experiments. Therefore, we tried to standardize our treatments, and used the same clone and parallels measured at the same period of the year in each experiment. However, it has to be noted that we observed slight seasonal variations within the essential oil composition and the chlorophyll contents (SPAD index values were not shown). Therefore, we decided to include and compare only data obtained in identical parallel experiments recorded in the same period of the year. This way, the control values, as well as the effect of the applied treatments, can be directly compared within each experiment but may not be compared among the different experiments.

Surprisingly, our data showed that the applied treatments induced no important alterations in the essential oil composition in any of the experimental settings. This is somewhat surprising, as literature data reported both quantitative and qualitative alterations in the essential oil (Chrysargyris et al., 2019). Based on our data we may speculate that the essential oil biosynthesis pathway of the “Moroccan” cultivar and clone used in our study may be less influenced by salt stress than other cultivars. It is also important to mention that we determined the essential oil composition in fully differentiated leaf pairs which had already well-developed glandular hairs containing essential oil, which is in contrast to literature data that either used whole plants for similar measurements including leaf primordia with developing glandular hairs (Aziz et al., 2008; Kasrati et al., 2014) or even flowers (Zekri et al., 2019). This might explain why we have not observed important changes in the essential oil composition. In our opinion, the statistically significant, but very small differences observed in some minor components, not specifically reported in the literature data before, may rather be related to decreased essential oil content (Aziz et al., 2008; Kasrati et al., 2014; Sarmoum et al., 2019) observed under stress conditions. Due to this decrease, some minor components go beyond the limits of the detection. This resulted in alterations in the relative distribution of the remaining detected components, with the strongest apparent effect observed in components present in very low amounts in the original sample.

Another major aim of our study was to improve our understanding of the potential effects of salinity and osmotic stress on chloroplast structure and activity in the leaves of intact, i.e., rooted plants. In this respect, our data showed that osmotic stress-induced a significant decrease in the chlorophyll content (SPAD index—Figure 9), in the RWC (Figure 11) and a slight, but significant decrease in photosynthetic activity (Figure 10) of the leaves, while the native organization of the chlorophyll-protein complexes (Figure 8) and chloroplast ultrastructure was almost unaffected (Figure 12) by the osmotic component of the salt stress.

Salinity has a negative impact on chlorophyll biosynthesis (Abdelkader et al., 2007) and also destabilizes pigment-protein complexes (Jaleel et al., 2008). In control and mildly stressed leaves, chlorophyll fluorescence emission bands corresponding to PSII core antenna (band maximum position at 685 and 695 nm) as well as to PSI light-harvesting complex (emission maximum at around 735 nm) were observed (Figures 5, 8), similarly to green leaves in the literature (Briantais et al., 1986). However, long-term, high concentration salt stress in the rootless plants, as well as the osmotic stress, had induced a strong decrease in the RWC values and alterations in the native chlorophyll-protein complexes, especially in the fluorescence of the PSI complex. Similarly to literature data, these changes were accompanied by decreased chlorophyll content (Li et al., 2015) and SPAD values (Choi and Chiang, 2015), while control SPAD values were similar to or slightly lower than data in the literature in mint species (Ronga et al., 2018; Yan et al., 2020). It must be noted that these values strongly vary among species, cultivars, developmental stages, as well as environmental conditions, e.g., light intensity. Therefore, the comparison was mostly possible using the same genotype and experimental settings as well as leaves of the same developmental stages. In dark-adapted leaves, the Qy dark values corresponded to the maximum light energy conversion efficiency of PSII. Qy dark values lower than 0.6 indicated serious disturbances of PSII activity as described in the literature data for other mint species under severe salinity stress (Li et al., 2015; Khalvandi et al., 2021).

We collected several pieces of information about the strong deleterious effect of salinity on the photosynthetic activity and chloroplast structure of spearmint. Our comparative investigations using isosmotic PEG solutions clearly outlined that (i) the loss of granum regularity (Figures 12, 13), (ii) increased electron-density of the stroma, (iii) and the appearance of the crystalline inclusions resembling electron-transparent spotted bodies, (iv) as well as the stronger decrease in physiological parameters (Qy values, SPAD index, RWC, organization of the chlorophyll-protein complexes; Figures 8–11), can be attributed to the specific ionic components of salt stress. These data are important since often in other crops, e.g., in fenugreek, the swelling of the thylakoid lumen and other ultrastructural alterations are attributed to the osmotic component of salinity (Evelin et al., 2013), while we had proved that it was not the case for spearmint plants used in this experiment. Similar spotted bodies sometimes defined as stroma centers have been described in several species under disturbances in ion homeostasis of the plastids (Solymosi and Bertrand, 2011, 2012). The loss of granum regularity observed by TEM (Figure 12) was also confirmed using for the first time SANS to analyze spearmint granum structure *in vivo* (Figure 13). Neutron scattering techniques are widely used for the characterization of the structure and dynamics of condensed matter. A key advantage of neutrons is their electric neutrality, allowing them to penetrate deeply into most materials, without affecting the mesoscopic organization of soft or liquid phase samples, such as biological membranes and tissues. Our data about the effect of abiotic stresses on the ultrastructural organization of grana indicated a substantial degree of flexibility in the membrane ultrastructure. In addition, they paved the way for further experiments to study the *in vivo* effects of combined stressors or different stresses (light or heat stress) on the organization and short- or long-term dynamic structural alterations of grana in spearmint. Also, the findings of this study outlined the clear biological limitations of experiments carried out on excised leaf segments or injured, e.g., freshly cut, plant organs.

Similarly to literature data, we used hydroponic-like salinity treatments, and concentrations, as well as treatment lengths, similar to those used by other authors (Gao et al., 2015b; Goussi et al., 2018; Chrysargyris et al., 2019). We are aware of the limitations of hydroponic cultures as well as of data obtained with freshly cut shoots. Pot experiments have lower reproducibility due to local variations and inhomogeneities in soil composition and structure, unequal salt accumulation in the pots, and a constantly increasing concentration of salt in the pots during treatment which make it difficult to precisely control salt concentrations. Due to extensive vegetative propagation of mint species by rhizomes, it is also highly difficult to grow mint in soil culture with a well-controlled root-to-shoot ratio. In addition, due to the above factors, deciphering the osmotic and specific ionic components of salt stress is not possible in pot experiments, because osmolarity cannot be controlled in them precisely. We observed a strong decrease in the chlorophyll content (SPAD index) already at relatively low concentration (25 mM NaCl) but longer treatments (2 weeks) (Figure 3). Similar observations have been described in other works (Chrysargyris et al., 2019). When the RWC values of the leaves decreased during various salinity stress treatments to one-third of their original values, i.e., 35–37%—Figures 2, 11; Supplementary Tables 1, 4), the activity of the photosynthetic apparatus was strongly disturbed (Figures 4, 10; Supplementary Tables 2, 3), and the relative chlorophyll contents significantly decreased (Figures 3, 9; Supplementary Tables 2, 3). Similar observations were made in maize under PEG and salinity treatment, which resulted in a decrease in the RWC, chlorophyll content, and decreased photosynthetic values (F_v_/F_m_) (Gao et al., 2015b). Cellular integrity was lost, but still, grana could be recognized in the TEM images (Figures 6B–D, 12C–E), and the essential oil composition of the leaves was not affected (Tables 1, 2). Indeed, it is important to note that chloroplast structure showed the swelling of the lumen only in case of the still greenish, but strongly wilted leaves treated for 2 weeks using 50 mM NaCl (Figure 6), while leaves or leaf regions that were brownish and fully dry did not exhibit swelling. The swelling of the thylakoid lumen (Salama et al., 1994; Yamane et al., 2008, 2012; Omoto et al., 2010; Evelin et al., 2013; Gao et al., 2015a; Goussi et al., 2018) is thought to be associated with oxidative stress and membrane damage (lipid peroxidation and the formation of different reactive oxygen species) (Miller et al., 2010; Oueslati et al., 2010; Yamane et al., 2012; Suo et al., 2017) and chloride influx from the stroma into the lumen (Bose et al., 2017). The observed alterations in plastoglobuli (increased number and size, changed electron-density; Figures 6, 12) may also reflect changes and potential degradation in the membrane components under stress (Hernández et al., 1995; Solymosi and Bertrand, 2012; Acosta-Motos et al., 2017). The presence of an electron-dense luminal substance (Keresztes and Sárvári, 2001) in spearmint thylakoids may be worth further investigations in order to understand its role in preserving granum structure in almost fully dried and highly stressed leaves (Figures 6, 7, 12). Similarly, it might be interesting to analyze whether the increased electron-density of the stroma under salt stress (Figures 6, 7, 12) may be accompanied by the accumulation of antioxidants or polyphenolic compounds (Oueslati et al., 2010; Chrysargyris et al., 2017, 2019).

On the other hand, our studies also outlined that at low concentrations (50 mM NaCl and especially below) the native organization of the chlorophyll-protein complexes of the photosynthetic apparatus was well retained (Figure 5) and plastid ultrastructure was also well preserved even despite moderately decreased RWC, photosynthetic activity and relative Chl contents (Figures 2–4). This is in agreement with no important ultrastructural changes being observed in fenugreek at 50 mM NaCl treatment, while larger alterations including swelling appearing at 100 and 200 mM NaCl (Evelin et al., 2013). It is also important to note that relatively low concentration (50 mM), but longer (2-week-long) stress applied to freshly cut shoots induced a stronger decrease in the photosynthetic activity (compare Qy values in Figures 4, 10; Supplementary Tables 2, 3) than high concentration (150 mM), shorter (1-week-long) stress applied on rooted plants. This outlines the important role of roots in the salt tolerance mechanism.

Our data showed that the “Moroccan” spearmint cultivar used in this work could tolerate cultivation in areas exposed to moderate osmotic and salt stress for relatively short periods (1 and 2 weeks). Furthermore, these stressors did not affect the essential oil composition of the fully developed leaves. However, above 50 mM NaCl concentrations, adventitious root formation and, thus, vegetative reproduction and clonal propagation were inhibited, and the water homeostasis, photosynthetic activity, and chloroplast structure of the leaves were also severely damaged.

## Data Availability Statement

The raw data supporting the conclusions of this article will be made available by the authors, without undue reservation.

## Author Contributions

The study was conceptualized and designed by KS. The indicated specific experiments and related data analyses were carried out by RÜ (SANS), FÁ, RO, and RH (SPAD, RWC, Qy values, and 77K fluorescence spectroscopy), KS (TEM and 77K fluorescence spectroscopy), BS-T, and AB (SPME and GC/MS). RO performed the RD calculations and analyses for TEM. Statistical analyses, Figures, and Tables were prepared by RO, FÁ, and KS. Data curation was made by RO, FÁ, RÜ, BS-T, and AB. The manuscript was mainly written by KS and RO but was complemented, edited, and approved by all authors.

## Funding

This work was supported by the János Bolyai Research Scholarship of the Hungarian Academy of Sciences (K.S.) and by the National Research, Development and Innovation Office (Grant OTKA FK 124748). RO and RH were financed by Stipendium Hungaricum Ph.D. Scholarship of the Tempus Public Foundation.

## Conflict of Interest

The authors declare that the research was conducted in the absence of any commercial or financial relationships that could be construed as a potential conflict of interest.

## Publisher's Note

All claims expressed in this article are solely those of the authors and do not necessarily represent those of their affiliated organizations, or those of the publisher, the editors and the reviewers. Any product that may be evaluated in this article, or claim that may be made by its manufacturer, is not guaranteed or endorsed by the publisher.
