# Supplementary material for: Salt Stress Affects Plastid Ultrastructure and Photosynthetic Activity but Not the Essential Oil Composition in Spearmint (Mentha spicata L. var. crispa “Moroccan”)
Source: Front Plant Sci. 2021 Oct 29;12:739467. doi: 10.3389/fpls.2021.739467 (PMC8586547; doi:10.3389/fpls.2021.739467)
Supplement: Supplementary file 1 [file Data_Sheet_1.docx]

Supplementary Material

**Supplementary Table S1. Relative water contents of the 3^rd^ and 4^th^ leaf pairs of freshly cut spearmint shoots before and after treatment with the given NaCl solutions in distilled water for 2 weeks at room temperature and ambient light conditions.** The mean values and the standard errors of the mean are indicated in the Table. Different letters indicate significant difference in each row according to 1-way-ANOVA followed by Tukey-Kramer multiple comparisons test (P < 0.05).

|  | **0 mM NaCl** | **0 mM NaCl** | **5 mM NaCl** | **25 mM NaCl** | **50 mM NaCl** |
| --- | --- | --- | --- | --- | --- |
|  | **Before** | **After** | **After** | **After** | **After** |
| **RWC**  (n=24-30) | 79.22±1.96a | 77.22±1.17a | 62.54±1.79b | 50.17±2.16c | 50.17±2.16c |

**Supplementary Table S2. Actual (Qy light) or maximal (Qy dark) quantum efficiency of photosystem II (measured in the light and 20-min dark-adapted states, respectively) and relative chlorophyll contents (measured as SPAD index) in the 3^rd^ and 4^th^ leaf pairs of freshly cut spearmint shoots before and after treatment with the given NaCl solutions in distilled water for 2 weeks at room temperature and ambient light conditions.** The mean values and the standard errors of the mean are indicated in the Table. Different letters indicate significant difference according to Kruskal-Wallis non-parametric ANOVA followed by Dunn’s multiple comparisons post hoc test (P < 0.05).

|  | **0 mM NaCl (H_2_O)** | | **5 mM NaCl** | | **25 mM NaCl** | | **50 mM NaCl** | |
| --- | --- | --- | --- | --- | --- | --- | --- | --- |
|  | **Before** | **After** | **Before** | **After** | **Before** | **After** | **Before** | **After** |
| **Qy light**  (n=23-33) | 0.78±0.00a | 0.78±0.00ab | 0.78±0.00ab | 0.76±0.00bc | 0.79±0.00a | 0.65±0.02cd | 0.78±0.01a | 0.47±0.03d |
| **Qy dark**  (n=24-36) | 0.82±0.01a | 0.80±0.00ab | 0.81±0.01a | 0.76±0.01bc | 0.82±0.00a | 0.68±0.02cd | 0.82±0.00a | 0.46±0.03d |
| **SPAD index**  (n=120-147) | 25.52±0.20a | 29.55±0.36b | 25.85±0.24a | 26.70±0.44a | 25.29±0.24a | 15.84±0.47a | 25.90±0.27c | 11.72±0.44c |

**Supplementary Table S3. Actual (Qy light) or maximal (Qy dark) quantum efficiency of photosystem II (measured in the light and 20-min dark-adapted states, respectively) and relative chlorophyll contents (measured as SPAD index) in 3^rd^ and 4^th^ leaf pairs of spearmint shoots before and after treatment with 0 mM NaCl (i.e. ‘H_2_O’, control, distilled water), 150 mM NaCl (in distilled water) and polyethylene glycol (PEG-6000, in distilled water, applied with equal osmolarity to the 150 mM NaCl solution) for 1 week at room temperature and ambient light conditions.** The mean values and the standard errors of the mean are indicated in the Table. Different letters indicate significant difference according to Kruskal-Wallis non-parametric ANOVA followed by Dunn’s multiple comparisons post hoc test (P < 0.05).

|  | **H_2_O** | | **150 mM NaCl** | | **Isosmotic PEG** | |
| --- | --- | --- | --- | --- | --- | --- |
|  | **Before** | **After** | **Before** | **After** | **Before** | **After** |
| **Qy light** (n=21-29) | 0.77±0.00a | 0.77±0.0a | 0.77±0.00a | 0.56±0.03b | 0.77±0.00a | 0.70±0.00b |
| **Qy dark** (n=16-24) | 0.80±0.00a | 0.79±0.00a | 0.80±0.00a | 0.52±0.00b | 0.80±0.01a | 0.70±0.01b |
| **SPAD index** (n=80-93) | 29.35±0.35a | 29.56±0.30a | 29.66±0.32a | 13.71±0.40b | 29.01±0.29a | 19.19±0.49c |

**Supplementary Table S4. Relative water contents of the 3^rd^ and 4^th^ leaf pairs of rooted spearmint plants before and after treatment with 0 mM NaCl (‘H_2_O’, control, distilled water), 150 mM NaCl (in distilled water) and polyethylene glycol (PEG-6000, in distilled water, applied with equal osmolarity to the 150 mM NaCl solution for 1 week at room temperature and ambient light conditions**. The mean values and the standard errors of the mean are indicated in the Table. Different letters indicate significant difference in each row according to 1-way-ANOVA followed by Tukey-Kramer multiple comparisons test (P < 0.05).

|  | **H_2_O** | **0 mM NaCl** | **150 mM NaCl** | **Isosmotic PEG** |
| --- | --- | --- | --- | --- |
|  | **Before** | **After** | **After** | **After** |
| **RWC** (n=18) | 82.72±0.83a | 83.05±0.73a | 37.21±2.89b | 59.30±2.18c |
